# Antioxidant Activity and Anti-Inflammatory Potential of *Rhododendron dauricum* Flowers via UPLC-Q-TOF-MS/MS Combined with Network Pharmacology and In Vitro Validation

**DOI:** 10.3390/plants15193052

**Published:** 2026-10-06

**Authors:** Yanan Liu, Xinyao Zhou, Yuanqi Duan, Qilin Shi, Yajie Liu, Zhengyu Hu, Man Li, Jinfeng Sun, Mei Jin, Tieqiang Zong, Wei Zhou, Gao Li

**Affiliations:** Key Laboratory of Natural Medicines of the Changbai Mountain, Ministry of Education, College of Pharmacy, Yanbian University, Yanji 133000, China; 18243390807@163.com (Y.L.); 18626718280@163.com (X.Z.); duanyuanqi163@163.com (Y.D.); 14717858088@163.com (Q.S.); 15703644309@163.com (Y.L.); huzhengyu1994@163.com (Z.H.); 15844274618@163.com (M.L.) jfsun@ybu.edu.cn (J.S.); jinmei198589@163.com (M.J.)

**Keywords:** *Rhododendron dauricum* L., flowers, UPLC-Q-TOF-MS/MS, network pharmacology, antioxidant, anti-inflammatory

## Abstract

*Rhododendron dauricum* L. flowers are highly valued as ornamental plants in northern Chinese horticulture, yet while its leaves are well-known for their antitussive and anti-asthmatic effects, the flowers themselves remain an underutilized resource. Oxidative stress and chronic inflammation are intimately intertwined pathological processes that mutually reinforce each other in a self-perpetuating cycle, and current clinical treatments for diseases related to oxidative stress and inflammation have become a significant focus of research. This study investigated the chemical composition and bioactivities of *R. dauricum* flower extract using UPLC-Q-TOF-MS/MS, network pharmacology, molecular docking, and in vitro assays. A total of 57 compounds were tentatively annotated in EFRD, with Morin, Quercetin 3-*O*-*α*-L-arabinopyranoside and Procyanidin B2 as candidate compounds prioritized through network analysis. Network prediction revealed key targets (STAT3, JUN, ESR1, CXCL8, PTGS2) and pathways (MAPK, AGE-RAGE, PPAR). In vitro, assays demonstrated that EFRD exhibited dose-dependent scavenging of DPPH, ABTS, and superoxide radicals, as well as a dose-dependent suppression of LPS-induced NO, TNF-α, and IL-6 release in RAW 264.7 macrophages, with IC_50_ values of 16.52, 29.91 and 72.09 μg/mL, respectively, indicating the most potent inhibition of NO. These findings demonstrate that *R. dauricum* flowers possess dual antioxidant and anti-inflammatory activities, providing a scientific basis for expanding their medicinal use.

## 1. Introduction

*Rhododendron dauricum* L. is a shrub species belonging to the genus *Rhododendron* in the family Ericaceae [1]. It is mainly distributed in Northeast China, Inner Mongolia, as well as northeast Asian regions including the Korean Peninsula and Russia, representing a characteristic plant resource in cold temperate zones [2,3]. As an excellent horticultural plant, *R. dauricum* features bright purplish-red flowers with a pleasant fragrance and elegant flower shape, serving as an important early-spring flowering shrub for landscaping in northern China. It is widely used in garden design and courtyard beautification. In parallel, *R. dauricum* exhibits strong ecological adaptability, making it a superior species for the restoration of degraded ecosystems and for soil and water conservation in northern regions. It possesses both ornamental and ecological value [4]. Additionally, *R. dauricum* also has a long history of application in the field of folk medicine. As the original plant of the traditional Chinese medicine “Manshanhong”, its leaves possess significant antitussive, expectorant, and anti-asthmatic effects, and have been traditionally used for the treatment of upper respiratory tract diseases [5,6]. In contrast, the flowers have traditionally been used mainly for ornamental purposes, and while a few ethnobotanical records mention their use for respiratory ailments [7], their ethnopharmacological relevance is considerably less documented than that of the leaves. Given the relatively limited ethnopharmacological documentation on the floral parts, this study was not designed to directly extend the traditional medicinal use of Manshanhong leaves; instead, it focused on a chemical and pharmacological exploration of the flowers.

In recent years, studies on the leaves of *R. dauricum* have become increasingly systematic, resulting in the isolation and identification of various components, including diterpenoids, flavonoids, phenolic acids, and volatile oils [8,9]. Furthermore, the antitussive, antioxidant, anti-asthmatic, and anti-inflammatory bioactivities of its leaf extracts have been thoroughly substantiated [10,11]. Clinically, this herbal medicine is widely used for the management of acute and chronic bronchitis, bronchial asthma, and other respiratory disorders. In contrast, research on the flowers of *R. dauricum* is relatively scarce, with only a few reports available. Among these studies, some researchers have focused on the pigment components in the flowers, investigating the extraction process and physicochemical properties of the flower pigments, and clarifying the application potential of their anthocyanin pigments [12]. Other studies have analyzed the volatile oil components of *R. dauricum* flowers, identifying dozens of volatile components, including *cis*-α-farnesene and (-)-bornyl acetate [13,14]. Several investigations have isolated diterpenoids from the flowers of *R. dauricum* and preliminarily found them to possess certain analgesic activity [15,16,17]. However, these investigations are incomplete, and a systematic analysis of the chemical composition of the flower extract of *R. dauricum* has not yet been conducted, with the vast majority of floral components remaining unstudied. This lack of research has led to the long-term underutilization of the abundant resource of *R. dauricum* flowers, resulting in significant waste of floral material and hindering the high-value development of this medicinal plant.

Accumulating evidence has demonstrated that oxidative stress and inflammation are intimately intertwined pathological processes that mutually reinforce each other in a self-perpetuating cycle [18]. Oxidative stress arises from an imbalance between the overproduction of reactive oxygen species (ROS) and the endogenous antioxidant defense system [19]. Excessive ROS not only directly damage macromolecules but also activate multiple transcription factors such as NF-κB, AP-1, and HIF-1α, which in turn upregulate pro-inflammatory cytokine expression, thereby initiating and amplifying inflammatory responses [20,21]. Conversely, inflammation perpetuates oxidative stress through a feedback mechanism: activated immune cells recruited to inflamed sites produce vast amounts of ROS, further exacerbating the oxidative burden in the local microenvironment [22]. Therefore, identifying natural bioactive components with dual antioxidant and anti-inflammatory properties from medicinal plants is of considerable interest for understanding their potential in vitro activities [23].

To address the above issues, this study adopted an integrated strategy of “component analysis—network prediction—preliminary in vitro evaluation”, aiming to characterize the chemical composition of *R. dauricum* flowers and preliminarily evaluate their in vitro antioxidant and anti-inflammatory activities, thereby providing a scientific basis for their resource development. Initially, UPLC-ESI-Q-TOF/MS spectrometry was used to comprehensively characterize the chemical constituents of the extract of *R. dauricum* flowers. Subsequently, network pharmacology was introduced to perform association analysis between the tentatively annotated chemical components and human disease targets, predicting potential health-related targets and providing research directions for subsequent in vitro experiments, thereby avoiding the blindness of traditional activity screening. Finally, based on the predicted directions, the antioxidant and anti-inflammatory activities of the *R. dauricum* flowers were experimentally validated using in vitro models, elucidating the bioactivity potential of *R. dauricum* flowers. We anticipate that this study will facilitate the development of *R. dauricum* flowers, transforming previously underutilized resources into functional products, and thereby enhancing the economic value of *R. dauricum*.

## 2. Results

### 2.1. UPLC-Q-TOF-MS/MS Analysis

**Figure 1:**
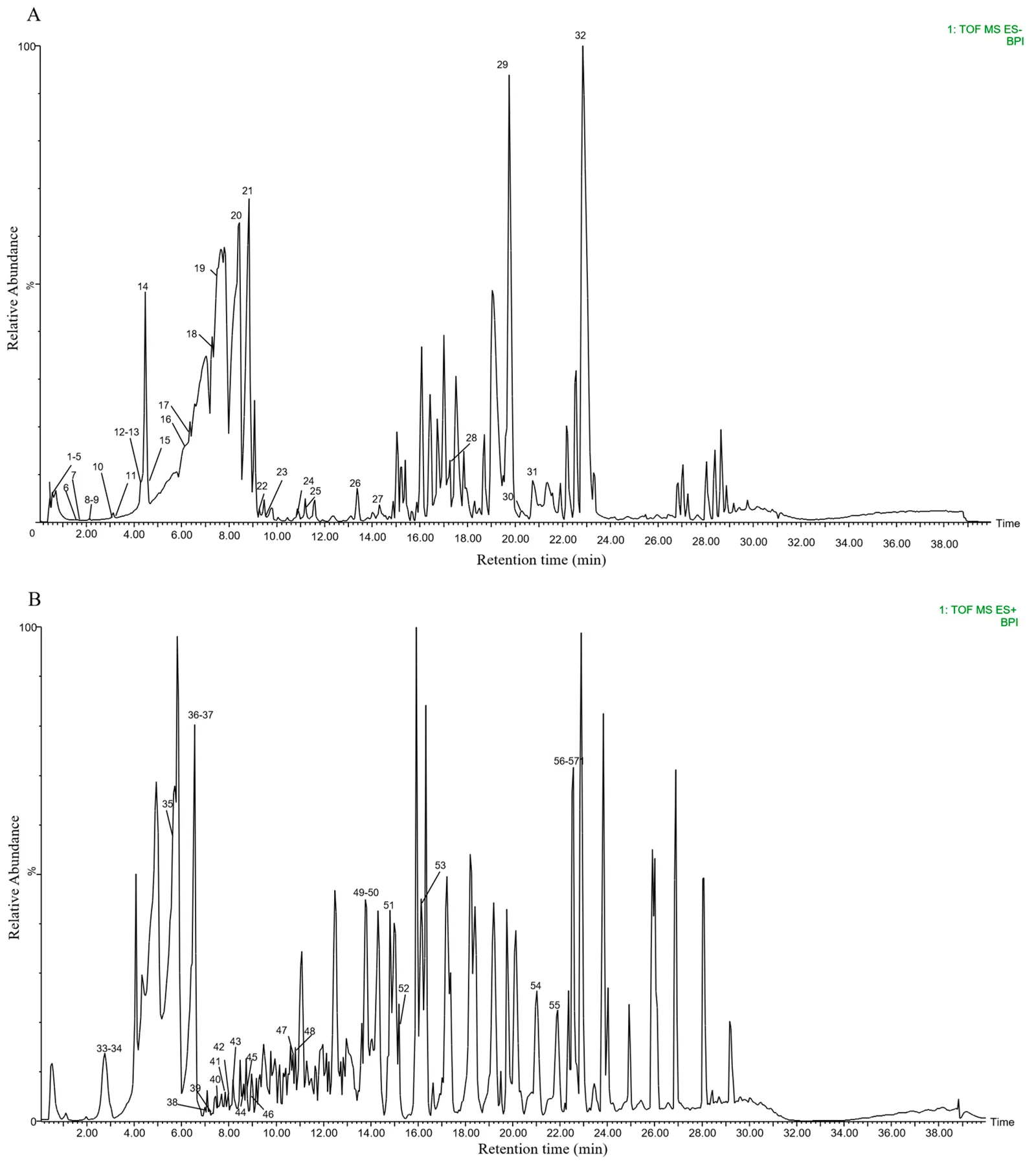
The chemical components in EFRD. Basic peak ion chromatogram (BPI) in ESI– mode (**A**), BPI in ESI+ mode (**B**).

**Figure 2:**
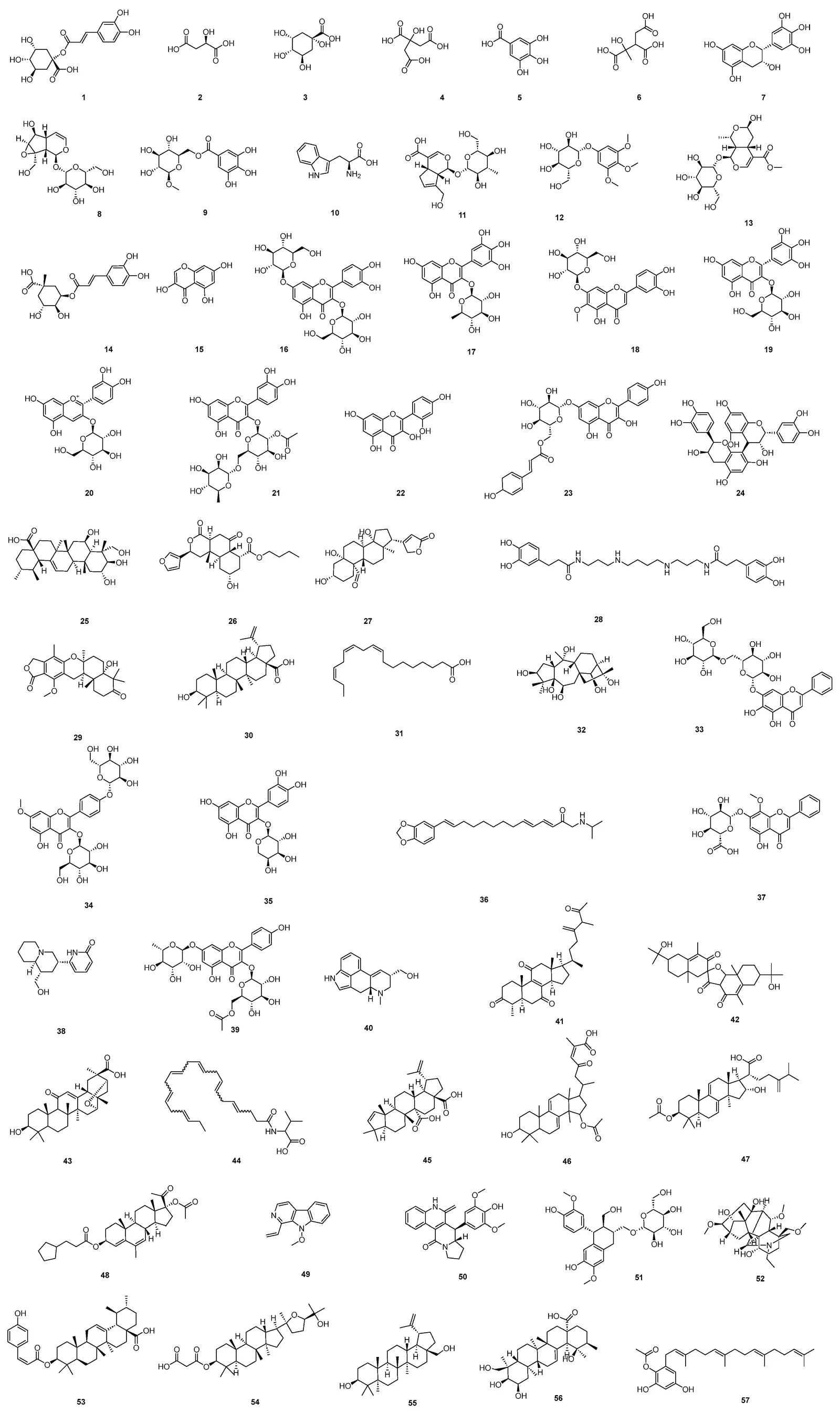
The chemical composition structure formula of the EFRD.

The chemical constituents of the EFRD were characterized using UPLC-Q-TOF-MS/MS. The base peak chromatograms (BPCs) of the extract in negative and positive ion modes are shown in Figure 1A and Figure 1B, respectively. A total of 57 compounds were tentatively annotated based on their accurate mass, retention time, and MS/MS fragmentation patterns, as summarized in Table 1. The structural formulas of all compounds are shown in Figure 2. The tentatively annotated compounds mainly include 18 terpenoids (including 11 triterpenes, four diterpenes, and three Iridoids), along with 16 flavonoids, six alkaloids, five organic acids, four phenylpropanoids, two aliphatics, three phenolics, two sterols, and one chromone. Under the current UPLC-Q-TOF-MS/MS conditions, grayanotoxins I, II, and III were not detected, whereas Andromedol was tentatively annotated at a relatively low level. Because authentic reference standards were not available and the analysis was untargeted, these findings do not prove the absence of grayanotoxins, and the safety of the flower extract remains to be established.

### 2.2. Network Pharmacology Analysis

#### 2.2.1. Collection of Active Compounds in EFRD and Target Proteins of Diseases

**Figure 3:**
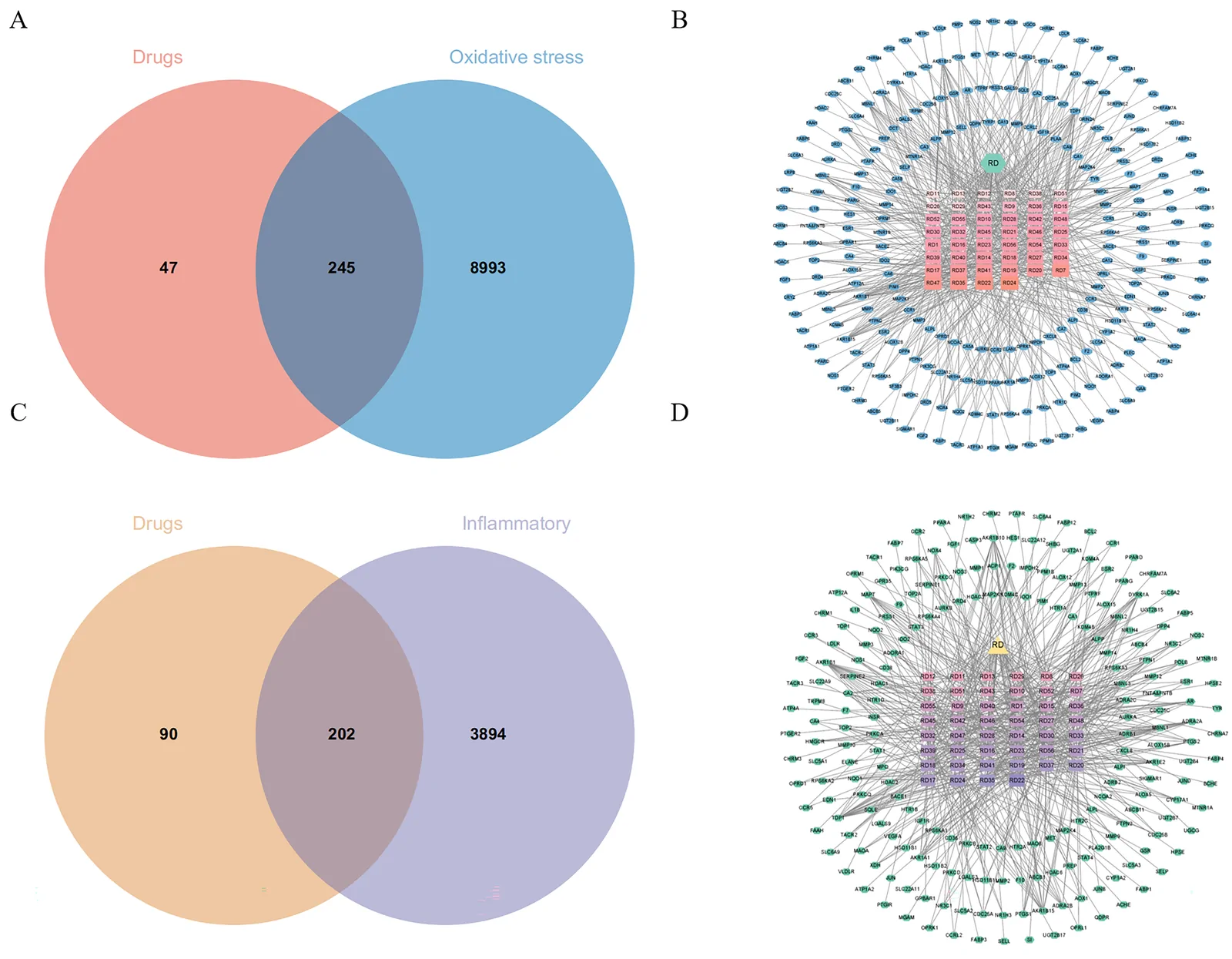
Target screening and ingredient–target regulatory network of EFRD: (**A**,**C**) the intersection of potential targets of active components in EFRD and disease targets; (**B**,**D**) active ingredient–target interaction network of the EFRD.

The potential targets of the active ingredients of EFRD were intersected with the targets associated with oxidative stress and inflammation respectively; 245 (Figure 3A) and 202 (Figure 3C) intersection targets were obtained in turn. The inclusion status, target counts, and reasons for exclusion for all 57 initially annotated compounds are summarized in Supplementary Table S1. The “active ingredient–target” interaction network (Figure 3B,D) was constructed to display the interaction relationship between the ingredients and the intersection targets. In the network, the nodes in the inner circle are the active ingredients of EFRD, and those of the outer circle are the intersection targets. The size and color depth of the nodes reflect the degree value; the higher the degree value, the more central the node is in the network. The results showed that components such as Morin, Quercetin 3-*O*-α-L-arabinopyranoside, Procyanidin B2 and Myricitrin ranked top, indicating that these components were candidate compounds prioritized through network analysis.

#### 2.2.2. Construction of Protein–Protein Interaction Network for Disease and EFRD

**Figure 4:**
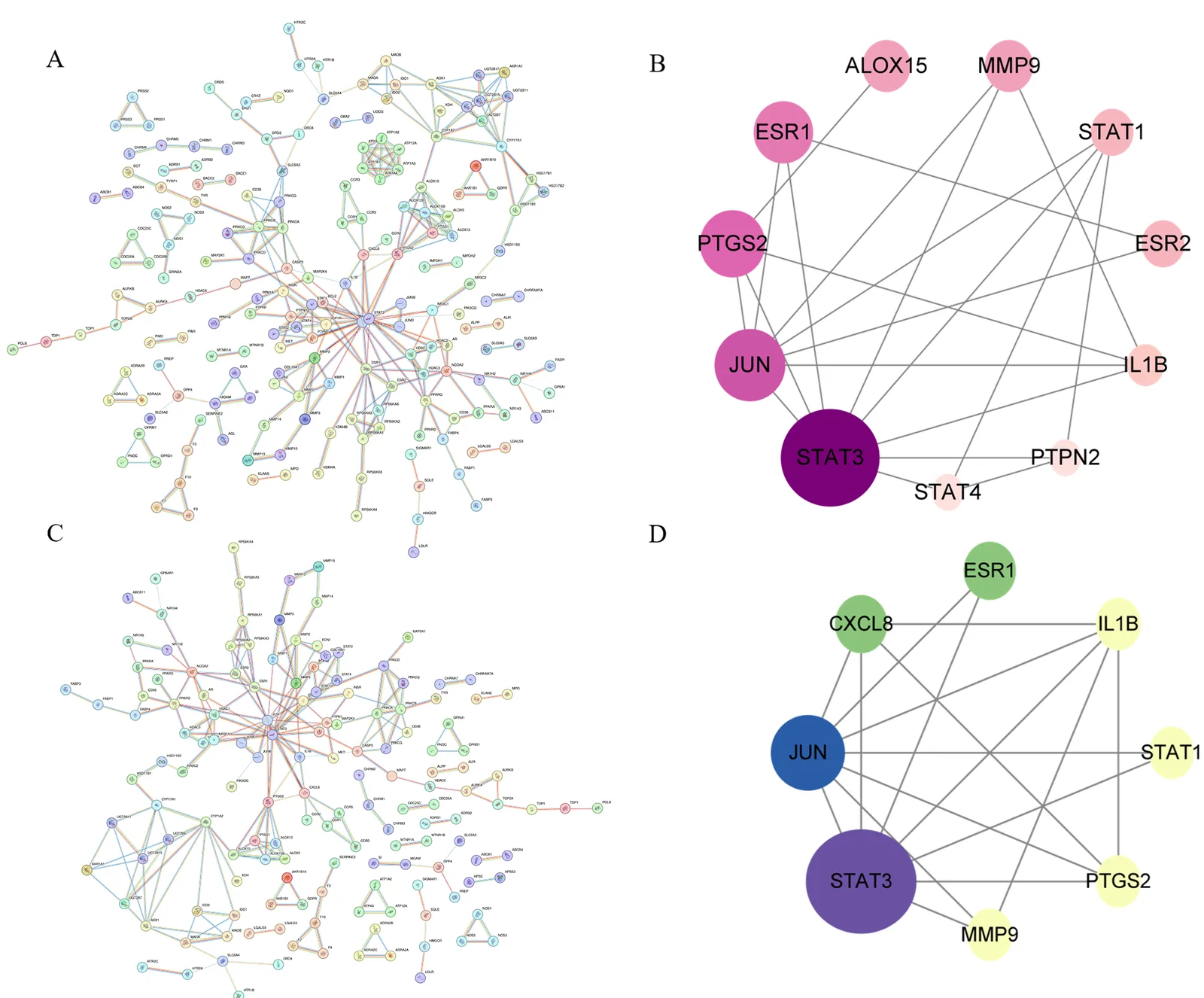
Protein–protein interaction (PPI) network analysis of overlapping targets of EFRD: (**A**,**C**) the PPI network diagram of target proteins; (**B**,**D**) the PPI network diagram of 15 core targets.

To identify the core targets through which EFRD exerts its biological effects, we imported the previously screened intersection targets related to oxidative stress and inflammation into the STRING platform, and the results are shown in Figure 4. The PPI networks respectively displayed 245 oxidative stress-related intersection targets (Figure 4A) and 202 inflammation-related intersection targets (Figure 4C), presenting the complex interactive regulatory relationships among the targets.

To further screen core regulatory targets, we extracted 15 core targets from each network and constructed a PPI network of the core targets (Figure 4B,D). The color depth of the nodes reflects the degree value of the targets, where darker colors indicate a more critical role of the target in the network. Notably, in the core networks related to oxidative stress (Figure 4B) and inflammation (Figure 4D), targets such as STAT3, JUN and ESR1 all showed high degree values, suggesting that they may be the common core regulatory nodes for EFRD to exert its antioxidant and anti-inflammatory biological effects. Furthermore, in the antioxidant and anti-inflammatory target networks, PTGS2 and CXCL8 exhibited the highest degree values, respectively, suggesting that these two may be the core regulatory targets mediating the antioxidant and anti-inflammatory pharmacological activities, respectively.

#### 2.2.3. GO and KEGG Pathway Enrichment Analysis

**Figure 5:**
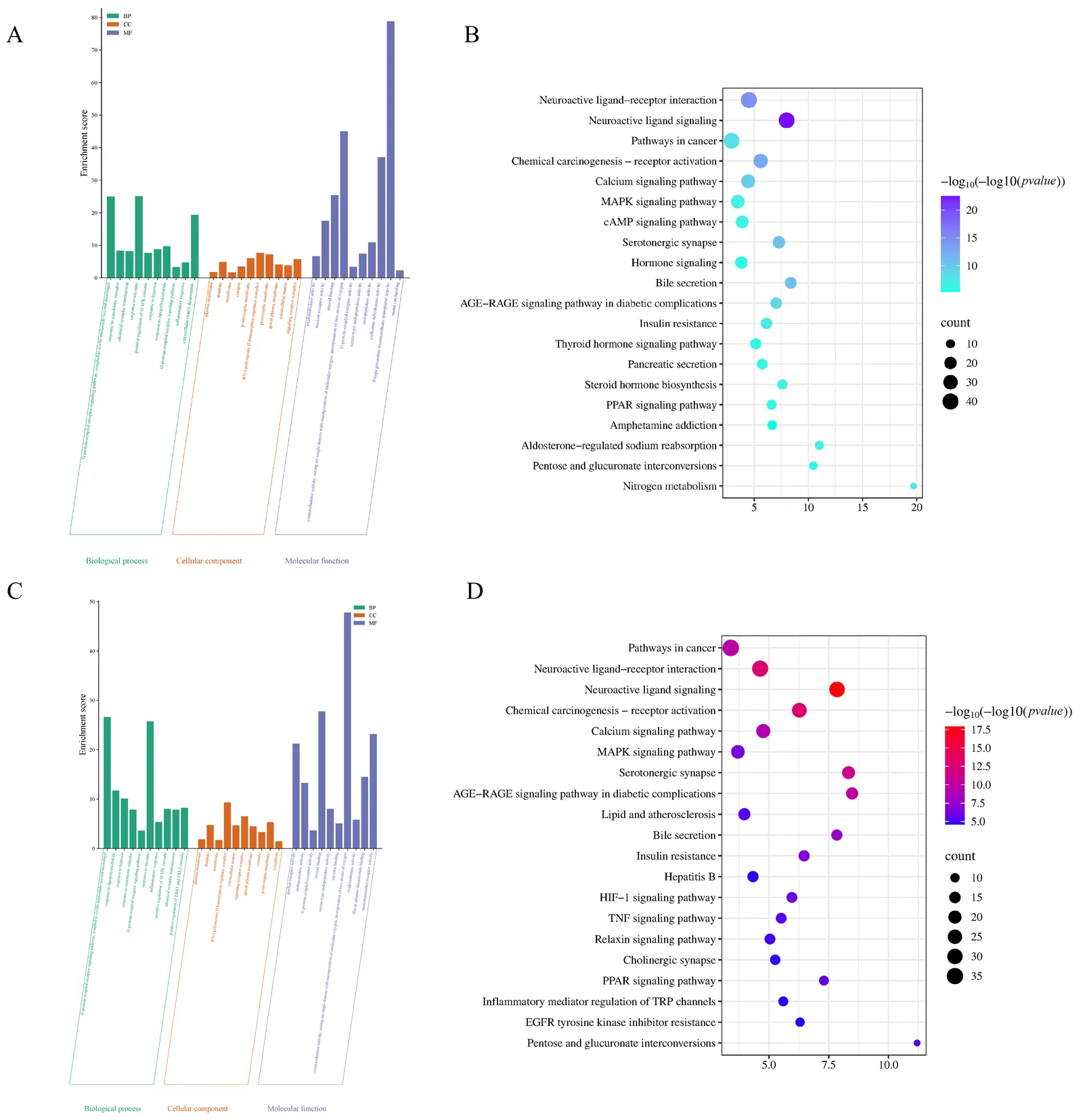
GO and KEGG pathway enrichment analysis of EFRD targets: (**A**,**C**) the GO analysis of the intersected genes (molecular function, cellular component, biological process); (**B**,**D**) KEGG pathway enrichment bubble diagrams.

GO functional enrichment and KEGG pathway enrichment analyses were performed on the intersection targets of oxidative stress and inflammation, respectively, and the results are shown in Figure 5. GO enrichment (Figure 5A,C) revealed that the biological processes (BPs) of the two types of targets were mainly enriched in the G protein-coupled receptor signaling pathway, response to lipopolysaccharide, response to hypoxia, response to xenobiotic stimulus, inflammatory response and other biological processes. In the cellular component (CC) analysis, these targets were largely associated with subcellular structures such as cell membrane, cytoplasm, signaling receptor complex, and synapse. The main enrichment terms of molecular function (MF) included oxidoreductase activity, enzyme binding, nuclear receptor activity, and steroid binding.

Based on the enrichment intensity and statistical significance, the top 20 key KEGG pathways were screened out. As shown in the KEGG pathway enrichment bubble plots for the two sets of targets (Figure 5B,D), pathways enriched in the oxidative stress targets (Figure 5B) include the MAPK signaling pathway, calcium signaling pathway, AGE-RAGE signaling pathway and PPAR signaling pathway, among others. Pathways enriched in the inflammation targets (Figure 5D) include the MAPK signaling pathway, AGE-RAGE signaling pathway, HIF-1 signaling pathway and PPAR signaling pathway, among others. Collectively, the two sets of targets associated with oxidative stress and inflammation share multiple core signaling pathways, such as the MAPK signaling pathway, the AGE-RAGE signaling pathway and the PPAR signaling pathway.

### 2.3. Molecular Docking of Active Compounds and Core Target Proteins

**Figure 6:**
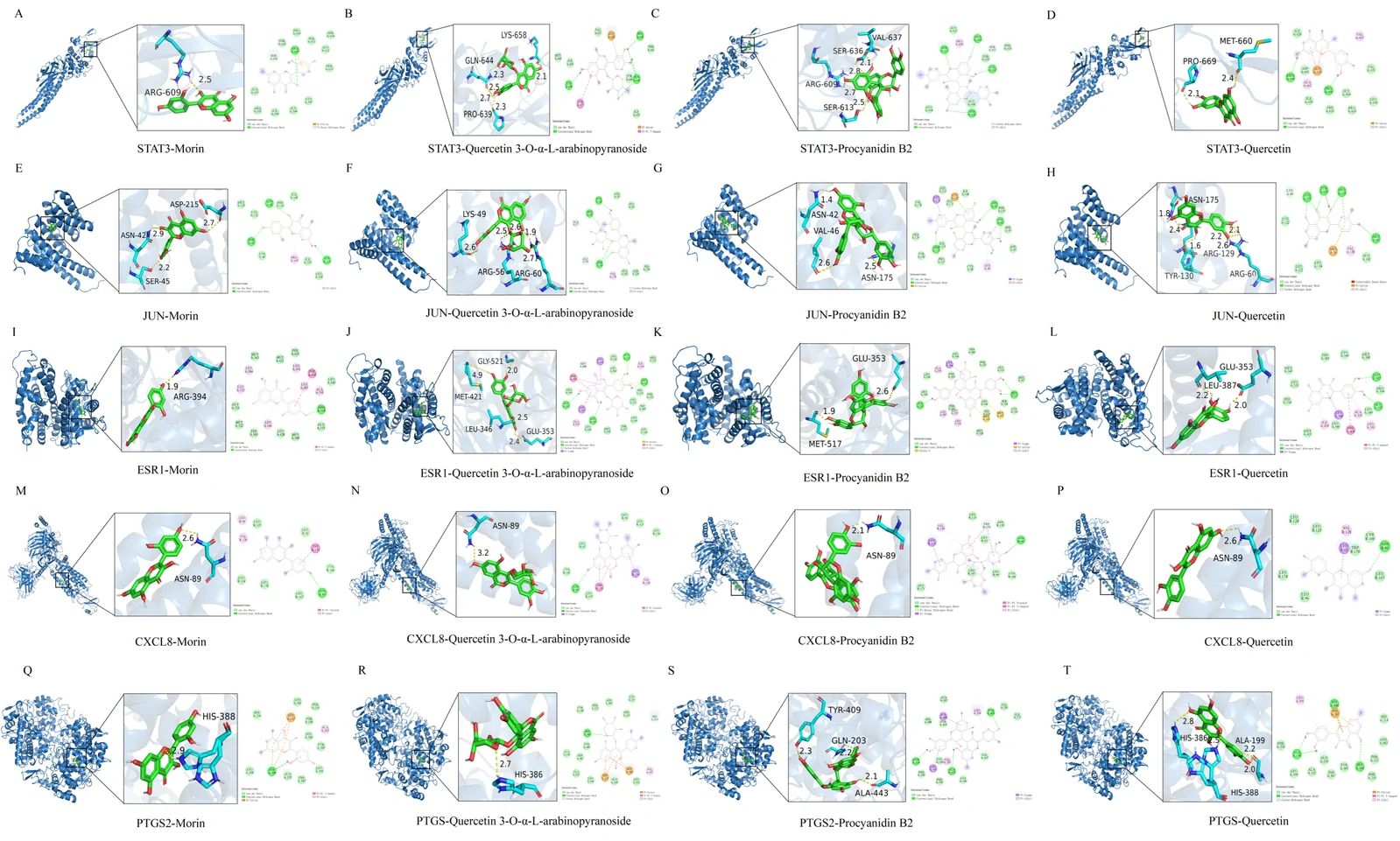
The molecular docking tests: (**A**–**D**) docking results of STAT3 combined with Morin, Quercetin 3-O-α-L-arabinopyranoside, Procyanidin B2, Quercetin, respectively; (**E**–**H**) docking results of JUN combined with the three ingredients above; (**I**–**L**) docking results of ESR1 combined with the three ingredients above; (**M**–**P**) docking results of CXCL8 combined with the three ingredients above; (**Q**–**T**) docking results of PTGS2 combined with the three ingredients above. Each panel contains the 3D overall protein structure, magnified binding pocket view and 2D ligand–protein interaction diagram.

Three major active ingredients tentatively annotated in EFRD (Morin, Quercetin 3-*O*-α-L-arabinopyranoside and Procyanidin B2) and five core targets screened in the previous study (STAT3, JUN, ESR1, CXCL8, PTGS2) were selected for molecular docking to predict their potential binding interactions. Quercetin, a compound widely used as a positive control in anti-inflammatory and antioxidant in vitro assays [23,24], was included as a reference ligand and docked against the same targets under identical parameters. The docking results are presented in Figure 6 and Table 2. The predicted binding free energies (ΔG) of the ingredient–target combinations ranged from −5.2 to −10.7 kcal/mol. The three active ingredients exhibited comparable or more favorable predicted binding affinities than quercetin for most targets. Notably, Procyanidin B2 showed the most favorable binding profile, with predicted affinities stronger than those of quercetin for most targets, particularly ESR1. All three active ingredients displayed more favorable predicted binding to STAT3 than quercetin, suggesting that STAT3 may be a common target for these compounds. For PTGS2, all compounds showed strong predicted binding, with quercetin being the most favorable. CXCL8 showed weaker predicted binding than the other targets, but its binding affinities were comparable to or more favorable than those of quercetin. These in silico results suggest that Procyanidin B2 may be a promising candidate for interacting with the core targets. However, these findings are hypothesis-generating and require experimental validation. In terms of binding modes, all active ingredients could precisely embed into the functional active pockets of target proteins and form stable interactions with key amino acid residues inside the pockets via hydrogen bonds, hydrophobic interactions, and other forces.

### 2.4. In Vitro Activity Verification

#### 2.4.1. Antioxidant Activity Analyses

**Figure 7:**
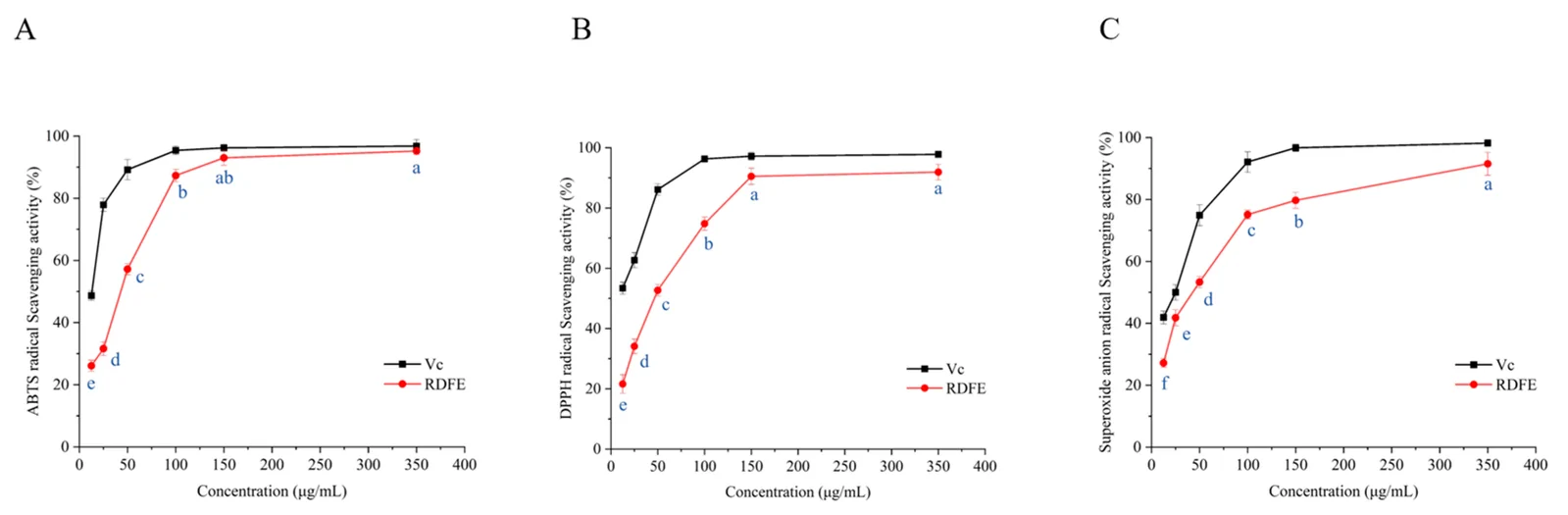
In vitro antioxidant assay analyses of EFRD and Vc. DPPH (**A**), ABTS (**B**), and superoxide anion (**C**). Data are mean ± SD (*n* = 3, triplicate). Values not sharing a common letter are significantly different (*p* < 0.05).

The antioxidant activity of EFRD was evaluated using DPPH, ABTS, and superoxide anion radical scavenging assays, with Vc serving as the positive control. As shown in Figure 7, both EFRD and Vc exhibited concentration-dependent radical scavenging activities across the tested concentration range. Vc demonstrated superior scavenging efficiency at low concentrations, whereas the activity of EFRD rose steadily with concentration and eventually reached a plateau. The IC_50_ values of EFRD were calculated to be 40.83 μg/mL (DPPH), 35.91 μg/mL (ABTS), and 33.5 μg/mL (superoxide anion), while those of Vc were 12.61 μg/mL (DPPH), 12.5 μg/mL (ABTS), and 19.74 μg/mL (superoxide anion). At the highest tested concentration, the maximum scavenging rates of EFRD in all three assays were comparable to those of Vc. Collectively, these results demonstrate that EFRD possesses potent in vitro antioxidant capacity.

#### 2.4.2. Anti-Inflammatory Activity Analyses

**Figure 8:**
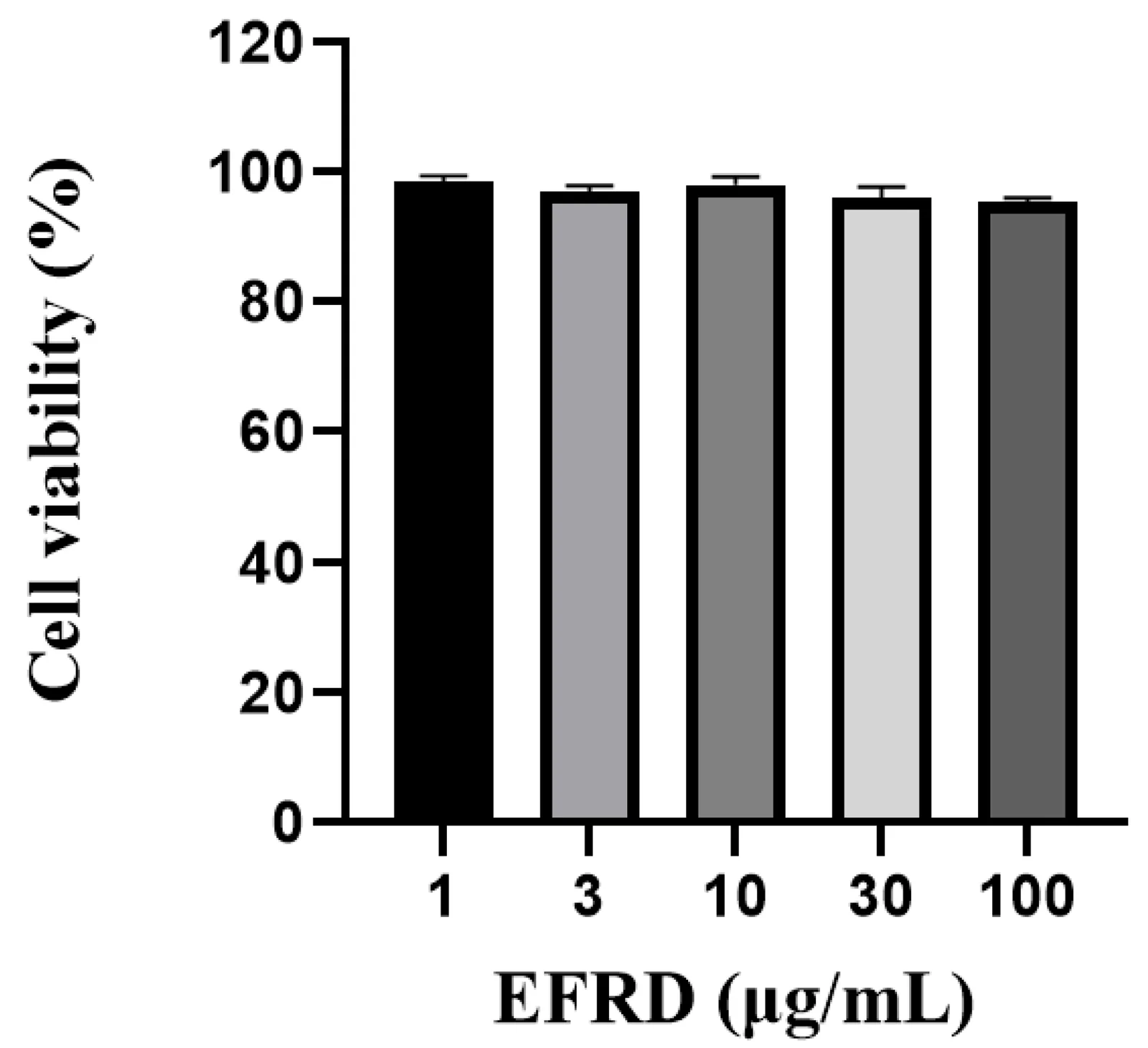
Cell viability of EFRD on RAW 264.7 cells. Data are mean ± SD (*n* = 3, triplicate).

An in vitro inflammatory model was established via LPS induction in RAW 264.7 cells. Using dexamethasone as the positive control agent, the expression levels of NO, IL-6, and TNF-α were quantified to assess the in vitro anti-inflammatory activity of EFRD, with the results summarized in Table 3. The MTT assay results showed that EFRD did not exhibit significant cytotoxic effects at concentrations ranging from 1 to 100 μg/mL (Figure 8). EFRD dose-dependently suppressed the LPS-stimulated release of the three pro-inflammatory mediators, with the most potent inhibitory effect on NO, followed by IL-6 and TNF-α. Compared with the dexamethasone positive control group, FRD exhibits a certain in vitro anti-inflammatory effect.

## 3. Discussion

*Rhododendron dauricum* L. is an important horticultural ornamental plant in northern China and the original plant of the traditional Chinese medicine “Manshanhong”. The antitussive, expectorant, and anti-asthmatic effects of its leaves have been thoroughly substantiated [25,26,27,28,29]. However, existing studies on the material basis and biological activities of *R. dauricum* flowers remain relatively scarce. Despite the abundant reserves of floral resources [30], they have long been used solely for ornamental purposes, representing a single form of utilization, and thereby restricting the comprehensive development and high-value utilization of this plant resource. The interplay between oxidative stress and chronic inflammation has been recognized as a reciprocally reinforcing cascade that drives the pathogenesis of various diseases [18]. Excessive ROS initiate inflammatory signaling and cytokine release, while inflammation conversely potentiates oxidative injury, together forming a self-amplifying cycle [31,32]. As such, the simultaneous modulation of oxidative stress and inflammation has become an actively pursued strategy in the search for novel therapeutic agents. Accordingly, there is a considerable demand for exploring multi-target, low-toxicity natural bioactive components from traditional medicinal plants for the prevention and treatment of these diseases. In the present study, we extended our investigation to the flowers of *R. dauricum*, a traditionally non-medicinal part of this medicinal plant. Through the integrated strategy of “component characterization—network prediction—preliminary in vitro evaluation”, we demonstrated that *R. dauricum* flowers exert significant dual antioxidant and anti-inflammatory activities. This work provides preliminary experimental evidence for tapping the potential value of *R. dauricum* flowers and expanding the scope of its medicinal resources. However, the specific targets and signaling pathways predicted by network pharmacology were not experimentally validated in this study, and further investigations are needed to confirm these mechanisms.

By means of UPLC-Q-TOF-MS/MS, various classes of chemical constituents from EFRD were tentatively annotated, including terpenoids, flavonoids, organic acids, phenylpropanoids, etc. Among them, Morin, Quercetin 3-*O*-α-L-arabinopyranoside, and Procyanidin B2 were tentatively annotated and predicted as candidate bioactive compounds through network analysis. This chemical profile shows partial differences from that of *R. dauricum* leaves. Accumulating studies have demonstrated that the leaves of *R. dauricum* are dominated by flavonoids represented by farrerol and quercetin [33,34,35], while the flowers are more abundant in organic acids and lignans [36]. This result suggests that the chemical composition of the flowers is not completely consistent with that of the leaves, which provides a material basis for their distinct biological activities.

Network pharmacology prediction results revealed that the active components of EFRD may potentially act on core targets including STAT3, JUN, ESR1, CXCL8 and PTGS2, and may be involved in multiple signaling pathways related to oxidative stress and inflammation, such as the MAPK, AGE-RAGE, and PPAR pathways. Among them, STAT3, as a key transcription factor in inflammatory pathways, is abnormally activated in airway epithelial cells and macrophages of asthmatic patients [37,38]. The MAPK signaling pathway is activated by reactive oxygen species (ROS) and inflammatory cytokines, and its downstream ERK, JNK and p38 cascades regulate the expression and release of pro-inflammatory mediators such as TNF-α, IL-6 and IL-1β [39,40]. The AGE-RAGE axis triggers oxidative stress and inflammatory injury via activation of RAGE, thereby promoting ROS generation and the release of inflammatory cytokines [41,42]. PPARs, particularly PPARγ, exert antioxidant and anti-inflammatory effects by upregulating antioxidant enzymes such as catalase, SOD-1 and heme oxygenase-1, and by suppressing the production of pro-inflammatory mediators [43,44,45]. Collectively, these three pathways each contribute to the regulation of oxidative stress and inflammatory responses. Molecular docking results suggested that candidate compounds such as Morin, Quercetin 3-*O*-α-L-arabinopyranoside and Procyanidin B2 might potentially bind to targets including STAT3, JUN, ESR1, CXCL8 and PTGS2. However, these docking results are hypothesis-generating in silico findings and require further experimental validation. The specific binding modes and regulatory effects of these compounds on the predicted targets remain to be confirmed.

In vitro experiments demonstrated that EFRD may interrupt the self-reinforcing vicious cycle between oxidative stress and inflammation via its dual antioxidant and anti-inflammatory effects. Free radical scavenging assays showed that EFRD exhibited significant scavenging capacity against DPPH, ABTS and superoxide anion radicals in a dose-dependent manner. This result suggests that EFRD can scavenge abundant ROS and attenuate the activation of inflammatory signaling pathways triggered by oxidative stress [46,47,48]. In the LPS-induced RAW 264.7 macrophage inflammatory model, EFRD inhibited the excessive release of NO and simultaneously downregulated the levels of pro-inflammatory cytokines, including TNF-α and IL-6. These findings indicate that EFRD possesses in vitro antioxidant and anti-inflammatory activities. However, whether these activities translate into modulation of specific disease mechanisms or therapeutic benefits requires further investigation.

One limitation of the present study is that we only verified the bioactivities of EFRD via in vitro experiments, and its in vivo efficacy has not been further validated. Nevertheless, we have demonstrated the antioxidant and anti-inflammatory activities of EFRD at multiple levels through network pharmacology combined with molecular docking and in vitro experiments, which provides a clear research direction for subsequent in vivo investigations. The safety profile and potential toxicity of EFRD were not systematically evaluated in this study. Although no obvious cytotoxicity was observed in RAW 264.7 cells at the tested concentrations, and the flowers were tentatively found to contain only a low level of Andromedol with no grayanotoxins I–III detected, their safety has not been established, and direct consumption of the flowers is not recommended. The presence of Andromedol and the previously reported cardiotoxicity of rhododendron in *R. dauricum* extracts highlight the need for dedicated toxicological assessment, including in vivo studies, before any clinical or therapeutic application can be considered. Further in-depth in vivo and in vitro studies are required to clarify its medicinal value in future work.

## 4. Materials and Methods

### 4.1. Reagents and Materials

The dried flowers of *R. dauricum* were obtained from Yanji City, Jilin Province, China, in October 2016 and authenticated by Prof. Gao Li. A voucher specimen (YB-RD-1610) was deposited at the College of Pharmacy, Yanbian University. ACQUITY UPLC^®^ I-Class (Waters Technology (Shanghai) Co., Ltd., Shanghai, China), Xevo ^®^ G2-S QTof (Waters Technology (Shanghai) Co., Ltd., Shanghai, China), centrifuge (Qingdao Jingcheng Instrument, GK-7F, Qingdao, China), 0.22 μm syringe filter (Pall Corporation, New York, NY, USA), analytical balance (0.0001 g, Mettler-Toledo, Greifensee, Switzerland), SB-800DTD ultrasonic cleaner (Ningbo Scientz Biotechnology Co., Ltd., Ningbo, China), Vortex-2 Genie vortex mixer (Scientific Industries, Bohemia, NY, USA), microplate reader (SH-1000, Corona Electric Co., Ltd., Hitachinaka, Japan) were used. Methanol (Shanghai Aladdin Biochemical Technology Co., Ltd., HPLC grade, Shanghai, China), acetonitrile (Aladdin, HPLC grade), water (ultrapure water, Wahaha, Hangzhou, China), formic acid (Shanghai Aladdin Biochemical Technology Co., Ltd., UltraPureChrom™, for HPLC, Shanghai, China), commercial specific complete DMEM (Invitrogen, Carlsbad, CA, USA), medium containing 10% fetal bovine serum (FBS, Hyclone, Logan, UT, USA), pancreatin, penicillin and streptomycin (Gibco, Carlsbad, CA, USA), dimethyl sulfoxide (DMSO) (SIGMA, CAS:67-68-5, Cat. NO. D2650-100ML), Dexamethasone and Lipopolysaccharide (Beyotime, Haimeng, China) were also utilized. The MTT (Sigma-Aldrich, St. Louis, MO, USA), DPPH (Shanghai YuanYe Biotechnology Co., Ltd., Shanghai, China), anhydrous ethanol (Anhui Sanxing Resin Technology Co., Ltd., analytical grade, Anhui, China), ABTS (Shanghai YuanYe Biotechnology Co., Ltd., analytical grade), K_2_S_2_O_8_ (Shanghai YuanYe Biotechnology Co., Ltd., analytical grade, Shanghai, China), Vc (Shanghai YuanYe Biotechnology Co., Ltd., analytical grade, Shanghai, China), Tris (Shanghai YuanYe Biotechnology Co., Ltd., analytical grade, Shanghai, China), pyrogallol (Shanghai YuanYe Biotechnology Co., Ltd., analytical grade, Shanghai, China) were also used.

### 4.2. Extract of the Flowers of R. dauricum (EFRD) Preparation

Dried flowers of *R. dauricum* (900.0 g) were finely ground and passed through an 80-mesh sieve (180 μm) to obtain a uniform powder. The powdered material was extracted by maceration with 80% ethanol (EtOH) at room temperature. The solid-to-liquid ratio was 2:1. The extraction was carried out for 3 weeks. During this period, the extract was collected once a week; after each collection, fresh 80% EtOH was added to the residue, and maceration was continued. After three weekly extractions, the three extracts were combined. The combined extract was filtered and then concentrated to dryness under reduced pressure using a rotary vacuum evaporator at 40 °C [49], yielding 102.3 g of extract. The extraction yield was 11.37% based on the dry weight of the starting material (102.3 g/900.0 g × 100%). The resulting *R. dauricum* flower extract was stored at 4 °C in a refrigerator and used for subsequent experiments.

### 4.3. UPLC-ESI-Q-TOF/MS

The EFRD (20 mg) was mixed with 20 mL of chromatographic-grade methanol and pure water (2:1), vortexed for 2 min, and ultrasonicated for 5 min. After filtration through a 0.22 μm membrane, 1.5 mL of the filtrate was transferred into a chromatographic vial for analysis [50]. UPLC-Q-TOF-MS analysis was performed using a Waters H-Class ultra-performance liquid chromatography system (Waters Technology (Shanghai) Co., Ltd.) coupled with a quadrupole-time-of-flight mass analyzer (Q-TOF, Waters Technology (Shanghai) Co., Ltd.). Chromatographic separation was conducted on a Waters BEH C18 column (1.7 μm, 2.1 × 50 mm, Waters Technology (Shanghai) Co., Ltd.). Gradient elution was performed using 0.1% formic acid (mobile phase A) and 0.1% formic acid in acetonitrile (mobile phase B) with the following program: 0–2 min, 5% B; 2–30 min, 5–98% B; 30–35 min, 98% B; 35–39 min, 98–5% B; 39–40 min, 5% B. The flow rate was maintained at 0.4 mL/min throughout the analysis. The column temperature was set at 40 °C, and the total run time was 40 min.

Mass spectrometry was performed using a Waters G2-XS Q-TOF mass spectrometer (Waters Technology (Shanghai) Co., Ltd.) equipped with an electrospray ionization (ESI) source, which was operated in both positive and negative ion modes. The MS parameters were set as follows: in positive ion mode, the capillary voltage was 2 kV, the ion source temperature was 110 °C, the desolvation temperature was 400 °C, the nitrogen flow rate was 800 L/h, the mass range was *m*/*z* 50–1200, and the collision energy was 20–40 V. In negative ion mode, the capillary voltage was 1.5 kV, whereas the ion source temperature, desolvation temperature, nitrogen flow rate, mass range, and collision energy were the same as those in positive ion mode.

The raw MS data were analyzed using Waters UNIFI software, and compound annotation was performed by searching the Waters UNIFI Traditional Chinese Medicine database. The mass tolerance was set to ±10 ppm, and the isotope pattern matching threshold was set to an intensity RMS ≥ 20%. Fragment matching required a mass error of ≤2 mDa and a retention time tolerance of ±0.1 min. Compounds matching these criteria were classified as MSI Level 2 (putatively annotated compounds) based on library MS/MS spectral matching. Compounds supported only by accurate mass and isotopic pattern without sufficient MS/MS confirmation were classified as MSI Level 3 (putatively characterized compound classes). Since no authentic reference standards were analyzed under identical conditions, none of the compounds were classified as MSI Level 1, and all annotations should be considered tentative.

This UPLC-Q-TOF-MS/MS analysis was intended solely for untargeted screening and tentative qualitative identification. No quantitative or semi-quantitative method validation was performed; therefore, calibration linearity, intra- and inter-day precision, accuracy, matrix recovery, LOD, and LOQ were not determined. The results should not be used for definitive quantitative conclusions.

### 4.4. Network Pharmacology

The active components tentatively annotated by UPLC-Q-TOF-MS/MS were selected and imported into the SwissTargetPrediction database (http://www.swisstargetprediction.ch/; version 2019) (accessed on 25 March 2026; probability > 0.1) and the Traditional Chinese Medicine Systems Pharmacology (TCMSP) database (https://tcmsp-e.com/; version 2.3) (accessed on 15 June 2026; DL > 0.18). Because the components tentatively annotated by UPLC-Q-TOF-MS/MS were mainly flavonoids and triterpenoids with low oral bioavailability, and because they may later be modified to improve bioavailability or administered by other routes, only DL > 0.18 was applied as the TCMSP screening criterion to avoid missing active components due to low oral bioavailability. After merging and deduplication, the potential targets of the components were obtained.

The GeneCards database (https://www.genecards.org/; version 5.26) (accessed on 28 June 2026; relevance score > 1) and the OMIM database (https://www.omim.org/) (accessed on 10 August 2026) were searched using the keywords “Oxidative stress” and “Inflammation” to obtain disease-related target genes. The UniProt database (https://www.uniprot.org/; release 2026_02) (accessed on 12 August 2026) was used to obtain standardized protein target information.

The Venny 2.1.0 online tool was used to generate a Venn diagram to obtain the intersecting genes between the active component targets of Rhododendron dauricum flowers and the disease targets. The drug–disease intersecting genes were uploaded to the STRING database platform (https://cn.string-db.org/) to construct a protein–protein interaction (PPI) network, with the species set to “Homo sapiens” and the minimum required interaction score set to 0.9 to ensure the reliability of this study [51]. After construction, the results were saved in TSV format. The TSV file was imported into Cytoscape 3.8.1 software, and the network was analyzed using the built-in application CentiScaPe 2.2 to screen key targets. During visualization, the size and color of the nodes reflected the Degree value. Nodes with a Degree value greater than or equal to twice the median were selected as hub nodes [52], and the genes corresponding to the hub nodes were the key target genes in the drug target-disease target network.

The obtained drug–disease intersecting genes were uploaded to the DAVID database (https://davidbioinformatics.nih.gov/). The gene identifier was set to OFFICIAL_GENE_SYMBOL, and the species was set to “Homo sapiens”. DAVID 6.8 was used for gene functional annotation. The obtained drug–disease intersecting genes were uploaded to the DAVID database (https://davidbioinformatics.nih.gov/) (accessed on 21 September 2026). The gene identifier was set to OFFICIAL_GENE_SYMBOL, and the species was set to “Homo sapiens”. DAVID 6.8 was used for gene functional annotation. DAVID uses Fisher’s exact test (hypergeometric distribution) to calculate the raw *p*-values for each GO term and KEGG pathway, and applies the Benjamini–Hochberg procedure to control the false discovery rate (FDR). The Benjamini-adjusted *p*-values and FDR values were directly obtained from DAVID. Both raw *p*-values and FDR values were reported. The detailed adjusted *p*-values/FDR values for GO and KEGG enrichment analyses are provided in Supplementary Tables S2–S5. The raw *p*-values were used for ranking and reference, whereas statistical significance was determined based on FDR < 0.05. The top 10 terms in biological process (BP), cellular component (CC), and molecular function (MF) from the GO functional annotation, as well as the top 20 KEGG pathways from the KEGG pathway analysis, were selected and imported into the BioInformatics Platform (http://www.bioinformatics.com.cn/) (accessed on 23 September 2026) for visualization. The biological processes and signaling pathways through which Rhododendron dauricum flowers exert their activities were predicted, providing direction for indicator selection and mechanism validation in subsequent in vitro experiments.

### 4.5. Molecular Docking

Based on the network pharmacology prediction results, the screened key targets and main active compounds were subjected to molecular docking. The two-dimensional structures of the active components were downloaded from the PubChem database (https://pubchem.ncbi.nlm.nih.gov/), imported into Chem3D 23.1.1 software to optimize the three-dimensional structures, and saved as mol2 format files. The protein receptor structures were obtained by screening high-resolution structures from the RCSB PDB database (https://www.rcsb.org/). The PDB IDs, selected protein chains, and resolutions for STAT3, JUN, ESR1, CXCL8 and PTGS2 are listed in Supplementary Table S6. PyMOL 2.6 software was used to remove redundant groups, such as water molecules, from the structures, and after pretreatment, the files were saved in PDB format.

AutoDockTools 1.5.6 software was used to preprocess the active components and protein receptor structures: atom types were assigned to small molecule ligands, and atomic charges were added. For the protein, atom types were assigned, atomic charges were added, and non-polar hydrogen atoms were removed. The files were then saved in PDBQT format. Subsequently, the receptor and ligand structure files were imported into AutoDock Vina, and the molecular docking grid box was defined. The ligands were set as flexible, while the receptor was set as rigid. The center coordinates and box dimensions of the active pocket were determined based on the binding site of the native ligand of the protein, with the grid box covering the entire active pocket region. The detailed grid-center coordinates, grid dimensions and the key AutoDock Vina parameters for STAT3, JUN, ESR1, CXCL8 and PTGS2 are provided in Supplementary Table S6. Quercetin was docked against the same core targets using identical parameters, and its predicted binding energies were compared with those of the tested active ingredients.

To validate the reliability and rationality of the docking parameters and protocol, re-docking was performed. The native ligand was extracted from the protein crystal structure and re-docked into the predefined active site of the target protein. The root-mean-square deviation (RMSD) between the re-docked ligand conformation and the native ligand conformation (crystal structure) was calculated. An RMSD < 2.0 Å was considered acceptable, indicating that the established docking protocol and parameters were reliable and could accurately reproduce the ligand–protein binding mode. All RMSD values were ≤2.0 Å, confirming the reliability of the docking protocol, and are summarized in Supplementary Table S6. Discovery Studio 2019 software and PyMOL 2.6 software were used for visualization, generating two-dimensional planar diagrams and three-dimensional binding mode diagrams.

### 4.6. Antioxidant Activity

#### 4.6.1. DPPH Radical Scavenging Activity

The DPPH radical scavenging activity of EFRD was determined according to a previously reported spectrophotometric method with minor modifications [53]. Briefly, a series of EFRD solutions at various concentrations were prepared, and vitamin C (Vc) was used as the positive control. Subsequently, 100 μL of each sample solution was mixed with an equal volume (100 μL) of freshly prepared DPPH ethanol solution (0.1 mM). The mixture was incubated in the dark at room temperature for 30 min, and the absorbance was measured at 517 nm using a microplate reader. The DPPH radical scavenging rate was calculated as the percentage inhibition according to the formula.

(1) $$\mathrm{DPPH}\;\mathrm{radical}\;\mathrm{scavenging}\;\mathrm{activity}\left(\%\right)=[1-\frac{\left(A1-A2\right)}{A0}]\;\times \;100\%$$

where A_0_ is the control group; A_1_ is the sample group; and A_2_ is the sample blank group.

#### 4.6.2. ABTS Radical Scavenging Activity

The ABTS radical scavenging activity of EFRD was determined according to a previously reported spectrophotometric method with minor modifications [54]. Briefly, the ABTS^+^ radical cation working solution was prepared by reacting 7 mM ABTS solution with 2.45 mM potassium persulfate in the dark at room temperature for 16 h. A series of gradient concentrations of EFRD were prepared, and Vc was employed as the positive control. Subsequently, 100 μL of each sample solution was mixed with an equal volume of diluted ABTS^+^ working solution. The mixture was incubated in the dark at room temperature for 6 min, and the absorbance was measured at 734 nm using a microplate reader. The ABTS radical scavenging percentage was calculated according to the corresponding formula:

(2) $$\mathrm{ABTS}\;\mathrm{radical}\;\mathrm{scavenging}\;\mathrm{activity}\left(\%\right)\;=\;[1-\frac{\left(A1-A2\right)}{A0}]\;\times \;100\%$$

where A_0_ is the control group; A_1_ is the sample group; and A_2_ is the sample blank group.

#### 4.6.3. Superoxide Anion Radical Scavenging Activity

The superoxide anion radical scavenging activity of EFRD was determined according to a previously reported pyrogallol autoxidation method with slight modifications [55]. Briefly, a series of EFRD solutions at various concentrations were prepared, and Vc was used as the positive control. The reaction mixture consisted of 100 μL of sample solution and 100 μL of Tris-HCl buffer (50 mM, pH 8.2). After pre-incubation at room temperature for 10 min, 100 μL of pyrogallol solution (2 mM) was added to initiate the autoxidation reaction. The mixture was incubated at room temperature for 4 min, and the absorbance was measured at 320 nm using a microplate reader. The superoxide anion radical scavenging rate was calculated as the percentage inhibition according to the formula:

(3) $$\mathrm{Superoxide}\;\mathrm{anion}\;\mathrm{radical}\;\mathrm{scavenging}\;\mathrm{activity}\left(\%\right)\;=\;[1-\frac{\left(A1-A2\right)}{A0}]\;\times \;100\%$$

where A_0_ is the control group; A_1_ is the sample group; and A_2_ is the sample blank group.

### 4.7. Anti-Inflammatory Activity

#### 4.7.1. Cell Viability Assay

Cell viability was measured by MTT assay. RAW 264.7 cells were purchased from BaiDi (Cat. No. C5142) and used between passages 2 and 7. Cells were cultured in DMEM supplemented with 10% fetal bovine serum and 1% penicillin/streptomycin at 37 °C in 5% CO_2_ and passaged every 2–3 days at 80–90% confluence. Routine mycoplasma testing was not performed, but cells were regularly examined and showed no signs of contamination. RAW 264.7 cells (1.2 × 10^5^cells/well) were seeded into 96-well plates for 24 h at 37 °C [56]. EFRD was dissolved in DMSO (final DMSO concentration ≤0.1%), and an equal-volume DMSO vehicle control was included. Cells were treated with EFRD at 1, 3, 10, 30, and 100 μg/mL for 24 h. Then, 20 μL of MTT was added and incubated for an additional 4 h. The absorbance was measured at 570 nm. MTT assay showed no cytotoxicity at 1–100 μg/mL, and these concentrations were used in subsequent assays. For LPS stimulation, a final concentration of 2 μg/mL was used. Dexamethasone (30 μM) was used as a positive control.

#### 4.7.2. Determination of NO Content in Cells

The content of nitric oxide (NO) in the cells was detected by the Griess method [57]. RAW 264.7 cells were seeded in 96-well plates at a density of 1.2 × 10^5^ cells per well and cultured for 24 h. The cells were then divided into the ctrl group, model group (LPS), positive drug group (LPS + dexamethasone) and EFRD treatment groups (LPS + EFRD at 1, 3, 10, 30, 100 μg/mL1, 3, 10, 30, 100 μg/mL), respectively. After 24 h of treatment, 50 μL of supernatant per well was aspirated and transferred to a new 96-well plate. Subsequently, 50 μL of Griess reagent I was added to each well and incubated at room temperature for 5 min, followed by the addition of 50 μL of Griess reagent II and further incubation for 5 min at room temperature, protected from light. The absorbance at a wavelength of 540 nm was measured by a microplate reader.

#### 4.7.3. Detection of Inflammatory Cytokines

The levels of TNF-α and IL-6 in cells were determined by ELISA [57]. One day in advance, 96-well plates were coated with TNF-α or IL-6 antibody. Each well was supplemented with 100 μL of antibodies, sealed and incubated overnight at 4 °C. On the next day, the supernatant was removed, and the plates were washed three times with 1 × PBS. Then, 200 μL of 1% BSA was added for blocking at room temperature for 1–2 h. Afterwards, 100 μL of standard substance or sample was added into each well (the drug administration treatment for TNF-α detection was the same as that for NO detection); for the drug administration treatment of IL-6 detection, all settings were consistent with NO detection except the EFRD group (LPS + EFRD, 1, 3, 10, 30, 100 μg/mL), and the mixture was incubated at room temperature for 2 h. Next, 100 μL of detection antibody was added and incubated at room temperature for 2 h. The plates were washed three times with 1 × PBST, then 100 μL of Streptavidin-HRP was added and incubated for 1 h away from light, followed by another three washes with 1×PBST. TMB chromogenic solution was added and reacted in the dark for 30 min. After the stop solution was added, the OD value was measured at 450 nm. For TNF-α detection, 50 μL of TMB and 25 μL of stop solution were used, while 100 μL of TMB and 50 μL of stop solution were applied for IL-6 detection.

### 4.8. Statistical Analysis

All experiments were performed with at least [*n* = 3] independent biological replicates. Data are presented as mean ± standard deviation (SD) or mean ± standard error of the mean (SEM), as indicated in the figure legends. Statistical analyses were performed using GraphPad Prism 9.0. Prior to parametric testing, the normality of data distribution was assessed using the Shapiro–Wilk test, and homogeneity of variance was assessed using Levene’s test. When these assumptions were met, one-way analysis of variance (ANOVA) followed by Tukey’s post hoc test was used for multiple comparisons. When variances were unequal, Welch’s ANOVA followed by Dunnett’s T3 post hoc test was used. When data were not normally distributed, the Kruskal–Wallis test followed by Dunn’s post hoc test was used. A *p*-value < 0.05 was considered statistically significant. IC_50_ values were calculated using GraphPad Prism 9.0. The log-transformed inhibitor concentrations were fitted to the normalized response by nonlinear regression using a four-parameter logistic (4PL) model (log[inhibitor] vs. normalized response, variable slope): Y = Bottom + (Top − Bottom)/(1 + 10^((LogIC50 − X) × HillSlope)^). IC_50_ was defined as the concentration producing 50% response between the upper and lower plateaus. The 95% confidence intervals were computed using the asymmetrical profile likelihood method.

## Figures and Tables

**Table 1:** Tentatively annotated of the chemical compounds of EFRD by UPLC-Q-TOF-MS/MS.

No.	tR (min)	Detected	Theoretical	Relative Molecular Mass	Deviation (ppm)	Molecular Formula	Daughter Ions (*m*/*z*)	Identification
1	0.52	353.0866	353.0872	354.0950	1.699296944	C_16_H_18_O_9_	353.0866 [M-H]^−^, 191.0568	1-Caffeoylqui nic acid
2	0.58	133.0138	133.0137	134.0215	−0.751802258	C_4_H_6_O_5_	133.0138 [M-H]^−^, 115.0034, 87.0078, 71.0127	D-Malic acid
3	0.63	191.0568	191.0555	192.0633	−6.804305555	C_7_H_12_O_6_	191.0568 [M-H]^−^, 85.0294	Quinic acid
4	0.73	191.0200	191.0191	192.0270	−4.711570728	C_6_H_8_O_7_	191.0200 [M-H]^−^, 173.0092, 85.0294	Citric acid
5	1.11	169.0139	169.0137	170.0215	−1.183336025	C_7_H_6_O_5_	169.0139 [M-H]^−^, 151.0034, 125.0266, 113.0276, 111.0085	Gallic acid
6	1.46	205.0364	205.0348	206.0426	−7.803553348	C_7_H_10_O7	205.0364 [M-H]^−^, 143.0365, 113.0254, 101.0253, 89.0243, 87.0097	2-Methylcitric acid
7	1.97	305.0667	305.0661	306.0739	−1.96678687	C_15_H_14_O_7_	305.0667 [M-H]^−^, 303.0551, 285.0421, 179.0334, 137.0233, 125.0221	(-)-Epigallocatechin
8	2.25	361.1170	361.1135	362.1212	−9.692243574	C_15_H_22_O10	361.1170 [M-H]^−^, 359.0993, 343.1048, 199.0553, 137.0257, 127.0326	Catalpol
9	2.35	345.0851	345.0822	346.0899	−8.403794806	C_14_H_18_O_10_	345.0851 [M-H]^−^, 315.0757, 285.0698, 225.0448, 183.0283, 169.0112, 161.0488	Methyl 6-*O*-galloyl-*β*-D-glucoside
10	2.90	203.0824	203.0821	204.0898	−1.477235069	C_11_H_12_N_2_O_2_	203.0824 [M-H]^−^, 203.0853, 159.0966, 142.0652, 116.0451	L-Tryptophan
11	3.64	373.1153	373.1135	374.1212	−4.824269291	C_16_H_22_O_10_	373.1153 [M-H]^−^, 211.0611, 123.0458	Geniposidic Acid
12	3.89	345.1194	345.1186	346.1263	−2.31804371	C_15_H_22_O_9_	345.1194 [M-H]^−^, 329.0896, 327.1076, 167.0350	3,4,5-Trimethoxyphenyl-*β*-D-glucopyranoside
13	4.17	405.1423	405.1397	406.1475	−6.417539431	C_17_H_26_O_11_	405.1423 [M-H]^−^, 373.1153, 243.0896, 179.0334	Morroniside
14	4.48	353.0904	353.0873	354.0950	−8.779698392	C_16_H_18_O_9_	353.0904 [M-H]^−^, 191.0568, 179.0361, 135.0468	Neochlorogenic acid
15	4.83	193.0150	193.0137	194.0215	−6.735273196	C_9_H_6_O_5_	193.0150 [M-H]^−^, 176.0138, 137.0257,	3,5,7-Trihydroxychromone
16	5.36	625.141	625.1405	626.1483	−0.7998202	C_27_H_30_O_17_	625.1410 [M-H]^−^, 463.0882, 462.0804	Quercetin 3,7-diglucoside
17	6.30	463.0882	463.0877	464.0954	−1.079709092	C_21_H_20_O_12_	463.0882 [M-H]^−^, 435.0933, 316.0257, 271.0277, 151.0037	Myricitrin
18	7.24	477.1049	477.1033	478.1111	−3.353571438	C_22_H_22_O_12_	477.1049 [M-H]^−^, 271.0243, 227.0350	Nepitrin
19	7.34	479.0852	479.0826	480.0903	−5.427039095	C_21_H_20_O_13_	479.0852 [M-H]^−^, 451.0882, 271.0243	Myricetin 3-*O*-glucoside
20	8.43	447.0928	447.0927	448.1005	−0.223667262	C_21_H_20_O_11_	447.0928 [M-H]^−^, 284.0334, 256.0388, 255.0309, 227.0361, 211.0405	Cyanidin 3-glucoside
21	8.73	651.1575	651.1561	652.1639	−2.150022091	C_29_H_32_O_17_	651.1567 [M-H]^−^, 301.0354, 217.0248	2″-*O*-Acetylrutin
22	9.26	301.0359	301.0348	302.0426	−3.654062587	C_15_H_10_O_7_	301.0359 [M-H]^−^, 300.0240, 271.0255, 257.0442, 243.0298, 178.9978, 151.0064	Morin
23	9.31	593.1324	593.1295	594.1373	−4.889320123	C_30_H_26_O_13_	593.1324 [M-H]^−^, 447.0933, 429.0827, 285.0405, 257.0455	Buddlenoid A
24	10.86	577.1375	577.1346	578.1424	−5.024824365	C_30_H_26_O_12_	577.1375 [M-H]^−^, 289.0728, 245.0840, 227.0350	Procyanidin B2
25	11.24	503.3394	503.3373	504.3450	−4.172152551	C_30_H_48_O_6_	503.3394 [M-H]^−^, 485.3217, 457.1043	Madecassic acid
26	13.36	417.1950	417.19133	418.1991	−8.796922985	C_23_H_30_O_7_	417.1957 [M-H]^−^, 399.1839, 359.1458, 301.1497	Diosbulbin H
27	14.30	403.2126	403.2121	404.2198	−1.240042151	C_23_H_32_O_6_	403.2126 [M-H]^−^, 385.2020, 359.2228	Cynotoxin
28	17.26	529.3044	529.3026	530.3104	−3.400701225	C_28_H_42_N_4_O_6_	529.3044 [M-H]^−^, 499.2150, 437.0045, 293.2159	kukoamine A
29	20.02	427.2121	427.2121	428.2198	0	C_25_H_32_O_6_	427.2121 [M-H]^−^, 393.1721, 367.1909	Austalide L
30	20.33	455.3556	455.3525	456.3603	−6.807912551	C_30_H_48_O_3_	455.3556 [M-H]^−^, 439.3594	Betulinic acid
31	20.43	277.2191	277.2168	278.2245	−8.296755464	C_18_H_30_O_2_	277.2191 [M-H]^−^, 216.8548, 116.9276	Linolenic acid
32	20.84	369.2240	369.2277	3702355	8.395916768	C_20_H_34_O_6_	269.2240 [M-H]^−^, 351.1986, 325.2202	Andromedol
33	2.65	595.1649	595.1663	594.1584	2.352283723	C_27_H_30_O_15_	595.1649 [M+H]^+^, 433.1125, 313.0700, 271.0657	Oroxin B
34	2.75	625.1744	625.1769	624.1690	3.99886816	C_28_H_32_O_16_	625.1744 [M+H]^+^, 463.1128, 301.0625	Complanatuside
35	5.67	435.0959	435.0927	434.0849	−7.354754516	C_20_H_18_O_11_	435.0959 [M+H]^+^, 303.0403, 225.0677,	Quercetin 3-*O*-α-L-arabinopyranoside
36	6.05	384.2508	384.2539	383.2460	8.067582398	C_24_H_33_NO_3_	384.2508 [M+H]^+^, 340.1938, 241.1275	Guineensine
37	6.20	461.1060	461.1084	460.1005	5.204849879	C_22_H_20_O_11_	461.1060 [M+H]^+^, 285.0893, 270.0759, 168.9703	Oroxindin
38	6.98	263.1745	263.1760	262.1681	5.699607867	C_15_H_22_N_2_O_2_	263.1745 [M+H]^+^, 245.1681, 150.1262	Mamanine
39	7.03	637.1761	637.1769	636.1690	1.255538297	C_29_H_32_O_16_	637.1761 [M+H]^+^, 491.1148, 145.0537	Kaempferol 3-*O*-(6″-acetyl-galactoside) 7-*O*-rhamnoside
40	7.64	255.1481	255.1497	254.1419	6.270828459	C_16_H_18_N_2_O	255.1481 [M+H]^+^, 237.1395, 226.1252, 170.0923, 166.0669	Isolysergol
41	7.69	469.2955	469.2954	468.2875	−0.213085404	C_29_H_40_O_5_	469.2955 [M+H]^+^, 451.3095, 433.2786, 423.3159, 405.2737, 397.2705, 377.2142, 323.1232	(4α,5α)-4-Methyl-3,7,11-trioxoergosta-8,24(28)-dien-26-oic acid
42	7.97	513.3232	513.3216	512.3137	−3.116954362	C_31_H_44_O_6_	513.3232 [M+H]^+^, 485.3260, 455.2794, 437.2670, 415.2440	Carindone
43	8.07	485.326	485.3267	484.3188	1.442327405	C_30_H_44_O_5_	485.3260 [M+H]^+^, 483.3125, 467.3156, 419.2954	Liquoric acid
44	8.37	428.3124	428.3165	427.3086	9.572360626	C_27_H_41_NO_3_	428.3124 [M+H]^+^, 410.3038, 386.2675, 283.3104, 281.2215	N-Docosahexaenoyl Valine
45	8.68	455.3143	455.3161	454.3083	3.9532975	C_29_H_42_O_4_	455.3143 [M+H]^+^, 437.3013, 429.2957, 425.2637	Ceanothenic acid
46	8.96	527.3389	527.3373	526.3294	−3.034111185	C_32_H_46_O_6_	527.3389 [M+H]^+^, 481.3391, 455.3143, 399.2883, 397.3112, 393.2784	(24*E*)-15alpha-Acetoxy-3alpha-hydroxy-23-oxo-7,9(11),24-lanostatrien-26-oic acid
47	10.70	527.3765	527.3737	526.3658	−5.309328091	C_33_H_50_O_5_	527.3765 [M+H]^+^, 449.3419, 353.2466	(3*β*,16*α*)-3-(Acetyloxy)-16-hydroxy-24-methylenelanosta-7,9(11)-dien-21-oic acid
48	10.81	511.3404	511.3424	510.3345	3.911273542	C_32_H_46_O_5_	511.3404 [M+H]^+^, 493.3399, 483.3170, 469.3354, 467.2713, 451.3225, 369.2422	(3*β*)-17-(Acetyloxy)-3-(3-cyclopentyl-1-oxopropoxy)-6-methylpregna-4,6-dien-20-one
49	13.57	225.1015	225.1028	224.0949	5.775139181	C_14_H_12_N_2_O	225.1015 [M+H]^+^, 209.0756, 199.0823, 195.0940, 193.0723	1-Ethenyl-9-methoxy-9H-pyrido[3,4-b]indole
50	13.62	407.1589	407.1607	406.1528	4.420858889	C_23_H_22_N_2_O_5_	407.1589 [M+H]^+^, 245.0623, 408.1177	Isaindigotidione
51	14.61	523.2128	523.2179	522.2101	9.74737294	C_26_H_34_O_11_	523.2128 [M+H]^+^, 343.1524, 329.2334, 327.1571, 325.1426, 315.1523, 219.1076, 15730635	Isolariciresinol 9′-*O*-beta-D-glucoside
52	15.24	438.2863	438.2856	437.2777	−1.597132098	C_24_H_39_NO_6_	438.2863 [M+H]^+^, 420.2306, 346.1124, 129.9998, 406.2591, 374.2343	Neoline
53	16.13	603.4044	603.4050	602.3971	0.994357024	C_39_H_54_O_5_	603.4044 [M+H]^+^, 585.3983, 423.2822, 317.2450, 315.2395	Xiongterpene
54	20.91	605.4049	605.4053	604.3975	0.660714401	C_35_H_56_O_8_	605.4049 [M+H]^+^, 587.3958, 501.3955, 499.3774, 457.3694	papyriferic acid
55	21.47	443.3850	443.3889	442.3810	8.795890019	C_30_H_50_O_2_	443.3850 [M+H]^+^, 425.3735, 413.3734, 381.3155, 371.3376, 249.2163, 191.1678, 135.1062, 133.0915	Betulin
56	22.71	505.3504	505.3529	504.3450	4.947038001	C_30_H_48_O_6_	505.3504 [M+H]^+^, 477.3598, 475.3495, 449.2812, 441.3339	Myrianthic acid
57	23.34	441.2970	441.3005	440.2926	7.931103636	C_28_H_40_O_4_	441.2970 [M+H]^+^, 423.2822, 413.3068, 399.2842, 285.2303, 317.1721, 245.2290	Bolegrevilol

**Table 2:** The binding free energies of the three main active components and five core targets in EFRD.

Compound	ΔG (kcal/mol)				
	STAT3	JUN	ESR1	CXCL8	PTGS2
Morin	−6.3	−6.4	−6.9	−5.2	−8.7
Quercetin 3-*O*-α-L-arabinopyranoside	−6.6	−6.3	−7.3	−5.4	−8.4
Procyanidin B2	−7.0	−7.2	−10.7	−6.2	−8.2
Quercetin ^a^	−6.1	−6.6	−7.4	−5.5	−9.2

^a^ Quercetin was used as a positive control.

**Table 3:** IC_50_ (95% CI) of dexamethasone and EFRD against LPS-induced inflammatory mediators in RAW 264.7 cells (*n* = 3, triplicate; 4PL model).

Compound	IC_50_ (μg/mL)		
	NO	IL-6	TNF-α
dexamethasone	3.29 ± 1.20	18.62 ± 1.77	16.56 ± 1.42
crude extract	16.52 ± 1.27	29.91 ± 2.36	72.09 ± 1.58

## Data Availability

The original contributions presented in this study are included in the article; further inquiries can be directed to the corresponding author.

## Supplementary Materials

The following supporting information can be downloaded at: https://www.mdpi.com/article/10.3390/plants15193052/s1. Table S1: Compound inclusion and target counts for network pharmacology analysis; Table S2: Top 10 GO enrichment data for oxidative stress-related targets; Table S3: Top 20 KEGG pathway enrichment data for oxidative stress-related targets associated with antioxidant activity; Table S4: Top 10 GO enrichment data for inflammation-related targets; Table S5: Top 20 KEGG pathway enrichment data for oxidative stress-related targets associated with Anti-inflammatory activity; Table S6: Grid-center coordinates, grid dimensions, and key AutoDock Vina parameters for STAT3, JUN, ESR1, CXCL8, and PTGS2.

## Abbreviations

The following abbreviations are used in this manuscript:

DPPH	1,1-Diphenyl-2-picrylhydrazyl
ABTS	2,2′-Azino-bis(3-ethylbenzothiazoline-6-sulfonic acid)
MTT	3-(4,5-Dimethylthiazol-2-yl)-2,5-diphenyltetrazolium bromide
EtOH	Ethanol
NO	Nitric oxide
TNF-α	Tumor necrosis factor-α
IL-6	Interleukin-6
BSA	Bovine serum albumin
STAT3	Signal transducer and activator of transcription 3
ESR1	Estrogen receptor 1
CXCL8	Interleukin-8, IL-8
PTGS2	Prostaglandin—endoperoxide synthase 2, COX2
JUN	Jun proto-oncogene, AP-1 transcription factor subunit
