# Supplementary material for: Antioxidant Activity and Anti-Inflammatory Potential of Rhododendron dauricum Flowers via UPLC-Q-TOF-MS/MS Combined with Network Pharmacology and In Vitro Validation
Source: Plants (Basel). 2026 Oct 6;15(19):3052. doi: 10.3390/plants15193052 (PMC13667099; doi:10.3390/plants15193052)
Supplement: Supplementary file 1 [file plants-15-03052-s001.zip › plants-4568189-supplementary.pdf]

Supplementary Data

Antioxidant Activity and Anti-inflammatory Potential of *Rhododendron dauricum* Flowers via UPLC-Q-TOF-MS/MS Combined with Network Pharmacology and In *Vitro* Validation

Yanan Liu[1], Xinyao Zhou[1], Yuanqi Duan[1], Qilin Shi[1], Yajie Liu[1], Zhengyu Hu[1], Man Li[1], Mei Jin[1], Jinfeng Sun[1], Tieqiang Zong[1,*], Wei Zhou[1,*], Gao Li[1,*]

[1] *Laboratory of Natural Medicines of the Changbai Mountain, College of Pharmacy, Yanbian University, Yanji 133002, P.R. China*

Yanan Liu: 18243390807@163.com

Xinyao Zhou: 18626718280@163.com

Yuanqi Duan: duanyuanqi163@163.com

Qilin Shi: 14717858088@163.com

Yajie Liu: 15703644309@163.com

Zhengyu Hu: huzhengyu1994@163.com

Man Li: 15844274618@163.com

Mei Jin: jinmei198589@163.com (M.J.)

Jinfeng Sun: jfsun@ybu.edu.cn

Note:

*Corresponding authors

E-mail addresses: 0000008134@ybu.edu.cn *Tel*: (+86) 0433-2732083 (TQ Zong)

E-mail addresses: zhouwei8452@163.com *Tel*: (+86) 0433-2436007 (W Zhou)

E-mail addresses: gli@ybu.edu.cn *Tel*: (+86) 0433-2436001 (G Li)

List of content

Table S1. Compound inclusion and target counts for network pharmacology analysis.

Table S2. Top 10 GO enrichment data for oxidative stress-related targets.

Table S3. Top 20 KEGG pathway enrichment data for oxidative stress-related targets associated with antioxidant activity.

Table S4. Top 10 GO enrichment data for inflammation-related targets.

Table S5. Top 20 KEGG pathway enrichment data for oxidative stress-related targets associated with Anti-inflammatory activity.

Table S6. Grid-center coordinates, grid dimensions, and key AutoDock Vina parameters for STAT3, JUN, ESR1, CXCL8, and PTGS2.

Table S1. Compound inclusion and target counts for network pharmacology analysis

No	Compound name	Merged unique targets after deduplication	Included in network analysis (Yes/No)	Reason for exclusion
**1**	1-Caffeoylquinic acid	AKR1B10, AKR1B1, AKR1B15, AKR1A1, AKR1E2, MMP2, MMP9, MMP12, MMP13, MMP1, MMP3, MMP10, MMP27, MMP20, TDP1	Yes	-
**2**	D-Malic acid	-	No	DL < 0.18
**3**	Quinic acid	-	No	DL < 0.18
**4**	Citric acid	-	No	DL < 0.18
**5**	Gallic acid	-	No	DL < 0.18
**6**	2-Methylcitric acid	-	No	DL < 0.18
**7**	(-)-Epigallocatechin	PTGS1, PTGS2, TOP2, NCOA2, CXCL8, CA12, CA1, CA2, CA3, CA4, CA6, CA5A, CA7, BACE1, CA9, CA13, TDP1, CA5B, CA14, BACE2	Yes	-
**8**	Catalpol	DPP4, BCL2, CASP3, MAPT	Yes	-
**9**	Methyl 6-*O*-galloyl-*β*-D-glucoside	SQLE, F10, PTPN2, PTPN1, TDP1, F2, F9, F7, LGALS9, LGALS3, LGALS9B	Yes	-
**10**	L-Tryptophan	CA1, CA2, ALPL, CA3, ALPI, CA5A, CA7, PLAA, CA13, ALPP, ALPPL2, IDO1, IDO2, TACR2	Yes	-
**11**	Geniposidic Acid	CA2, F2, MAPT	Yes	-
**12**	3,4,5-Trimethoxyphenyl-*β*-D-glucopyranoside	TYR, TYRP1, DCT, TDP1	Yes	-
**13**	Morroniside	CA2, DPP4, MAPT	Yes	-
**14**	Neochlorogenic acid	PTGS2, AKR1B10, AKR1B1, AKR1B15, AKR1A1, AKR1E2, MMP2, MMP9, MMP12, MMP13, MMP1, MMP3, MMP10, MMP27, MMP20, ELANE	Yes	-
**15**	3,5,7-Trihydroxychromone	CDK5R1&CDK5, MAOA, MAOB, PTGS1, PTGS2, DYRK1A, AURKA, PIM1, AURKB, AURKC, PIM3, PIM2, INSR, IGF1R, MET	Yes	-
**16**	Quercetin 3,7-diglucoside	ADRA2A, AKR1B1, ADRA2C, NOX4, TDP1, AKR1B15, AKR1B10, AKR1A1, DYRK1A, ADRA2B, AKR1E2, MBNL1, MBNL2, MBNL3, NQO2	Yes	-
**17**	Myricitrin	TOP2, MPO, ADRA2A, AKR1B1, ADRA2C, NOX4, TDP1, AKR1B15, AKR1B10, AKR1A1, DYRK1A, ADRA2B, AKR1E2, NQO2, NQO1, MBNL1, MBNL2	Yes	-
**18**	Nepitrin	CA2, AKR1B1, AKR1B15, AKR1B10, TDP1, KDM4A, KDM4B, KDM4C, ADORA1, XDH, AOX1, ADRA2A, ADRA2C, NOX4, AKR1A1, DYRK1A	Yes	-
**19**	Myricetin 3-*O*-glucoside	TOP2, PTPN1, ADRA2A, AKR1B1, ADRA2C, NOX4, TDP1, AKR1B15, AKR1B10, AKR1A1, DYRK1A, ADRA2B, AKR1E2, NQO2, NQO1, MBNL1, MBNL2	Yes	-
**20**	Cyanidin 3-glucoside	CA2, TOP2, CD38, ADRA2A, AKR1B1, ADRA2C, NOX4, TDP1, AKR1B15, AKR1B10, AKR1A1, DYRK1A, ADRA2B, AKR1E2, NQO2, NQO1, MBNL1	Yes	-
**21**	2"-*O*-Acetylrutin	AKR1B1, AKR1B15, AKR1B10, ADRA2A, ADRA2C, MBNL1, DYRK1A, ADRA2B, MBNL2, MBNL3, RPS6KA3, RPS6KA5, RPS6KA4, RPS6KA2, RPS6KA1	Yes	-
**22**	Morin	PTGS1, AR, PPARG, PTGS2, DPP4, PIK3CG, TOP1, EDN1, CD36, DIO1, GSR, PIP4K2A, BATF3, ESR1, ESR2, SLC22A12, GPR35, TDP1, SLC22A10, SLC22A25, SLC22A9, SLC22A24, SLC22A11, CYP1A2, ALOX5, HSD17B1, AKR1B1, ALOX15	Yes	-
**23**	Buddlenoid A	XDH, AOX1, KDM4A, KDM4B, KDM4C, AKR1B1, AKR1B15, AKR1B10, TDP1, ADORA1, RPS6KA3, RPS6KA5, RPS6KA4, RPS6KA2, RPS6KA1	Yes	-
**24**	Procyanidin B2	MMP14, MAP2K1, PTGS2, PRKCA, PRKCB, MAP2K4, MMP2, MMP9, CA12, CA1, CA2, CA3, CA4, CA6, CA5A, CA7, BACE1, CA9, CA13, TDP1, CA5B	Yes	-
**25**	Madecassic acid	AKR1B10, AKR1B15, AKR1A1, AKR1B1, AKR1E2, MAPT, TDP1, PTPN2, PTPN1, HSD11B1, HSD11B1L, PLA2G1B, POLB, PTPRF, ACP1	Yes	-
**26**	Diosbulbin H	OPRM1, OPRD1, OPRK1, OPRL1, TDP1	Yes	-
**27**	Cynotoxin	NR3C2, TDP1, PTAFR, ATP12A, ATP1A1, ATP1A3, ATP4A, ATP1A2, ATP1A4, CDC25A, CDC25B, CDC25C, PPARG, PPARD, PPARA, MAPT	Yes	-
**28**	kukoamine A	OPRM1, OPRD1, OPRK1, OPRL1, QDPR, MTNR1A, MTNR1B, GPR50, CCR1, CCR2, CCR3, CCR5, CCRL2, SELL, SELP	Yes	-
**29**	Austalide L	FNTA&FNTB, IMPDH2, IMPDH1, SERPINE1, SERPINE2	Yes	-
**30**	Betulinic acid	AKR1B10, POLB, AKR1B1, SAE1&UBA2, AKR1B15, AKR1A1, AKR1E2, MAPT, NR1H4, HSD11B1, HSD11B1L, TOP2A, CDC25A, CDC25B, CDC25C	Yes	-
**31**	Linolenic acid	-	No	DL < 0.18
**32**	Andromedol	AKR1B10, NR1H3, UGT2B7, AR, CDC25B, CDC25A, SHBG, ESR2, ESR1, CA2, GRIN2A, CA4, TRPM8, GPBAR1	Yes	-
**33**	Oroxin B	KDM4A, KDM4B, KDM4C, AKR1B1, AKR1B15, AKR1B10, ADORA1, TDP1, XDH, AOX1, DYRK1A, MBNL1, MBNL2, MBNL3, ADRA2A	Yes	-
**34**	Complanatuside	TOP2, AKR1B1, NQO2, AKR1B15, AKR1B10, DYRK1A, NQO1, ADRA2A, ADRA2C, NOX4, TDP1, AKR1A1, ADRA2B, AKR1E2,	Yes	-

		MBNL1, MBNL2		
**35**	Quercetin 3-*O*-α-L-arabinopyranoside	PTGS2, PTPN1, TOP2, DPP4, AKR1B1, AKR1B15, AKR1B10, ADRA2A, ADRA2C, NOX4, TDP1, AKR1A1, DYRK1A, ADRA2B, AKR1E2, NQO2, NQO1, MBNL1, MBNL2	Yes	-
**36**	Guineensine	ADRB2, MAOB, MAOA, HDAC3, HDAC1, HDAC2, HDAC6, SIGMAR1, MBNL1, MBNL2, MBNL3, MAPT	Yes	-
**37**	Oroxindin	CA2, PTPN1, AKR1B1, AKR1B15, AKR1B10, ADORA1, TDP1, XDH, AOX1, KDM4A, KDM4B, KDM4C, PTGS2, PTGS1, RPS6KA3, RPS6KA5, RPS6KA4	Yes	-
**38**	Mamanine	CCR1、CCR2、CCR3、CCR5、CCRL2	Yes	-
**39**	Kaempferol 3-*O*-(6''-acetyl-galactoside) 7-*O*-rhamnoside	RPS6KA3, RPS6KA5, RPS6KA4, RPS6KA2, RPS6KA1, RPS6KA6, DYRK1A, AKR1B1, AKR1B15, AKR1B10, ADRA2A, ADRA2C, MBNL1, ADRA2B, MBNL2	Yes	-
**40**	Isolysergol	MBNL1, MBNL2, MBNL3, PLEC, DRD2, ADRB2, ADRB1, HTR1A, DRD1, DRD4, DRD5, HTR1D, HTR1B, HTR2A, HTR2C	Yes	-
**41**	(4α,5α)-4-Methyl-3,7,11-trioxoergosta-8,24(28)-dien-26-oic acid	NOS2, NOS3, NOS1, AR, CDC25A, CDC25B, NR3C1, SHBG, CYP17A1, NR3C2, SIGMAR1, MBNL1, MBNL2, MBNL3, FGF1, FGF2, VEGFA	Yes	-
**42**	Carindone	NR3C2, ACHE, BCHE, POLB, POLA1, CYP17A1, CRYZ, NOS2, NOS3, NOS1, FNTB&FTA, F2, AR, HMGCR	Yes	-
**43**	Liquoric acid	ALOX5, PREP, ALOX15B, ALOX12B, ALOX15, ALOX12, ALOXE3, F2, PRSS1, CTRC, PRSS2, PRSS3, CELA2A, CELA2B, CELA3B	Yes	-
**44**	N-Docosahexaenoyl Valine	-	No	TMSP: Not included in the database; SwissTargetPrediction: Null Results
**45**	Ceanothenic acid	AR, FAAH, FABP3, FABP1, FABP4, FABP5, FABP12, FABP7, PMP2, FABP9, FABP6, POLB, PTGER2, PTGIR, CDC25A	Yes	-
**46**	(24*E*)-15alpha-Acetoxy-3alpha-hydroxy-23-oxo-7,9(11),24-lanostatrien-26-oic acid	HMGCR, MAPT, TACR2, SLC6A2, SLC6A4, SLC6A3, TACR1, TACR3, SLC6A9, SLC6A7, SLC6A14, SLC6A5, CYP17A1, AR, TDP1, HPSE, HPSE2	Yes	-
**47**	(3*β*,16*α*)-3-(Acetyloxy)-16-hydroxy-24-methylenelanosta-7,9(11)-dien-21-oic acid	NR3C1, NR3C2, AR, HMGCR, PTPN2, PTPN1, HSD11B1, HSD17B2, HSD11B2, HSD11B1L, F2, FNTB&FTA, CYP17A1, MAPT, TACR2, STAT3, STAT1, STAT2, STAT4	Yes	-
**48**	(3*β*)-17-(Acetyloxy)-3-(3-cyclopentyl-1-oxopropoxy)-6-methylpregna-4,6-dien-20-one	MAPT, NR3C1, NR3C2, AR, IL1B, HMGCR, SF3B3, JUN, JUNB, JUND, PTGS2, PTGS1, MAOA, MAOB	Yes	-
**49**	1-Ethenyl-9-methoxy-9H-pyrido[3,4-b]indole	-	No	TMSP: Not included in the database; SwissTargetPrediction: probability > 0.1
**50**	Isaindigotidione	-	No	TMSP: Not included in the database; SwissTargetPrediction: Null Results
**51**	Isolariciresinol 9'-*O*-beta-D-glucoside	HES1, HES4, ESR1, ESR2, SLC5A1, SLC5A2, SLC5A11, SLC5A4, SLC5A10, SLC5A3, SLC5A9	Yes	-
**52**	Neoline	CHRNA7, CHRFAM7A, CHRM2, CHRM4, CHRM5, CHRM1, CHRM3, GBA, MGAM, GAA, SI, AGL, UGCG, GBA2	Yes	-
**53**	Xiongterpene	-	No	TMSP: Not included in the database; SwissTargetPrediction: Null Results
**54**	papyriferic acid	MBNL1, MBNL2, MBNL3, PRKCG, PRKCB, PRKCA, PRKCQ, PRKCD, ABCB1, ABCB11, ABCB4, ABCB5, PPM1B, PTPN1, PPM1A	Yes	-
**55**	Betulin	TDP1, LDLR, VLDLR, LRP8, UGT2B7, UGT2B11, UGT2B17, UGT2B4, UGT2B10, UGT2B15, UGT2A3, UGT2B28, UGT2A1, NR1H2, NR1H3	Yes	-
**56**	Myrianthic acid	PTPN2, PTPN1, AKR1B10, AKR1B15, AKR1A1, AKR1B1, AKR1E2, HSD11B1, HSD11B1L, MAPT, TDP1, PLA2G1B, POLB, PTPRF, ACP1	Yes	-
**57**	Bolegrevilol	-	No	TMSP: Not included in the database; SwissTargetPrediction: probability > 0.1

Table S2. Top 10 GO enrichment data for oxidative stress-related targets

Category	Term	Count	P-Value	Benjamini	Fold Enrichment	Bonferroni	FDR	Fisher Exact
GOTERM_BP_DIRECT	G protein-coupled receptor signaling pathway, coupled to	17	3.16E-18	8.28E-15	24.94	8.28E-15	7.6E-15	9.04E-20

GOTERM_BP_DIRECT	cyclic nucleotide second messenger response to xenobiotic stimulus	27	1.93E-16	2.53E-13	8.36	5.81E-13	2.32E-13	2.1E-17
GOTERM_BP_DIRECT	chemical synaptic transmission	24	2.11E-14	1.84E-11	8.20	5.52E-11	1.69E-11	2.34E-15
GOTERM_BP_DIRECT	response to nicotine	13	6.77E-14	4.43E-11	25.12	1.77E-10	4.07E-11	1.88E-15
GOTERM_BP_DIRECT	positive regulation of MAPK cascade	22	1.17E-12	5.98E-10	7.68	3.05E-09	5.49E-10	1.39E-13
GOTERM_BP_DIRECT	response to hypoxia	20	1.37E-12	5.98E-10	8.80	3.59E-09	5.49E-10	1.4E-13
GOTERM_BP_DIRECT	response to lipopolysaccharide	18	4.89E-12	1.83E-09	9.70	1.28E-08	1.68E-09	4.49E-13
GOTERM_BP_DIRECT	G protein-coupled receptor signaling pathway	43	1.42E-11	4.65E-09	3.29	3.72E-08	4.27E-09	4.16E-12
GOTERM_BP_DIRECT	inflammatory response	28	4.22E-11	1.23E-08	4.76	0.000000111	1.13E-08	8.39E-12
GOTERM_BP_DIRECT	extracellular matrix disassembly	11	1.77E-10	4.24E-08	19.37	0.000000464	0.000000039	7.18E-12
GOTERM_CC_DIRECT	plasma membrane	128	6.79E-14	2.25E-11	1.78	2.25E-11	1.92E-11	3.75E-14
GOTERM_CC_DIRECT	dendrite	30	4.01E-12	6.63E-10	4.88	1.33E-09	5.67E-10	7.78E-13
GOTERM_CC_DIRECT	membrane	121	9.31E-11	1.03E-08	1.67	3.08E-08	8.78E-09	5.51E-11
GOTERM_CC_DIRECT	synapse	33	1.8E-09	0.000000149	3.47	0.000000596	0.000000127	4.97E-10
GOTERM_CC_DIRECT	postsynaptic membrane	18	8.53E-09	0.000000565	6.04	0.00000282	0.000000483	1.32E-09
GOTERM_CC_DIRECT	RNA polymerase II transcription regulator complex	14	3.67E-08	0.00000202	7.68	0.0000121	0.00000173	4.38E-09
GOTERM_CC_DIRECT	presynaptic membrane	14	7.85E-08	0.00000371	7.20	0.000026	0.00000317	0.00000001
GOTERM_CC_DIRECT	apical plasma membrane	21	0.000000245	0.0000101	4.08	0.000081	0.00000866	0.000000057
GOTERM_CC_DIRECT	extracellular matrix	22	0.000000326	0.000012	3.84	0.000108	0.0000103	8.14E-08
GOTERM_CC_DIRECT	signaling receptor complex	15	0.000000378	0.0000125	5.75	0.000125	0.0000107	6.15E-08
GOTERM_MF_DIRECT	oxidoreductase activity	25	5.76E-13	3.99E-10	6.64	3.98E-10	3.33E-10	8.03E-14
GOTERM_MF_DIRECT	nuclear receptor activity	12	6.18E-11	2.14E-08	17.52	4.27E-08	1.79E-08	2.83E-12
GOTERM_MF_DIRECT	steroid binding	10	1.12E-10	2.58E-08	25.44	7.73E-08	2.16E-08	3.14E-12
GOTERM_MF_DIRECT	oxidoreductase activity, acting on single donors with incorporation of molecular oxygen, incorporation of two atoms of oxygen	8	1.5E-10	2.59E-08	45.06	0.000000104	2.17E-08	1.68E-12
GOTERM_MF_DIRECT	G protein-coupled receptor activity	37	2.8E-10	3.88E-08	3.38	0.000000194	3.24E-08	7.97E-11
GOTERM_MF_DIRECT	serine-type endopeptidase activity	18	3.48E-10	4.02E-08	7.43	0.000000241	3.36E-08	4.3E-11
GOTERM_MF_DIRECT	endopeptidase activity	14	4.61E-10	4.56E-08	10.93	0.000000319	3.81E-08	3.7E-11
GOTERM_MF_DIRECT	carbonate dehydratase activity	8	8.22E-10	7.12E-08	37.11	0.000000568	5.95E-08	1.31E-11

GOTERM_MF_ DIRECT	P-type potassium transmembrane transporter activity	6	1.83E-09	0.000000141	78.85	0.00000127	0.000000118	3.91E-12
GOTERM_MF_ DIRECT	metal ion binding	55	6.45E-09	0.000000447	2.30	0.00000446	0.000000374	2.73E-09

Table S3. Top 20 KEGG pathway enrichment data for oxidative stress-related targets associated with antioxidant activity.

Term	Count	P-Value	Benjamini	Fold Enrichment	Bonferroni	FDR	Fisher Exact
Neuroactive ligand signaling	38	3.31E-23	9.38E-21	7.99	9.38E-21	6.26E-21	3.46E-24
Neuroactive ligand-receptor interaction	40	1.96E-15	2.78E-13	4.52	5.66E-13	1.85E-13	4.41E-16
Chemical carcinogenesis - receptor activation	29	1.78E-13	1.68E-11	5.59	5.03E-11	1.12E-11	2.81E-14
Serotonergic synapse	20	2.12E-11	1.5E-09	7.28	5.99E-09	0.000000001	2.47E-12
Bile secretion	18	2.73E-11	1.54E-09	8.37	7.72E-09	1.03E-09	2.69E-12
Calcium signaling pathway	27	2.74E-10	1.29E-08	4.45	7.77E-08	8.65E-09	5.62E-11
AGE-RAGE signaling pathway in diabetic complications	17	1.64E-09	6.62E-08	7.04	0.000000463	4.42E-08	1.99E-10
Pathways in cancer	37	1.01E-08	0.000000358	2.91	0.00000286	0.000000239	3.29E-09
Insulin resistance	16	3.87E-08	0.00000122	6.14	0.0000109	0.000000812	5.51E-09
Nitrogen metabolism	8	6.23E-08	0.00000176	19.70	0.0000176	0.00000118	1.89E-09
MAPK signaling pathway	25	0.000000174	0.00000438	3.49	0.0000491	0.00000292	4.63E-08
Aldosterone-regulated sodium reabsorption	10	0.000000186	0.00000438	11.01	0.0000525	0.00000292	1.31E-08
Steroid hormone biosynthesis	12	0.000000367	0.00000798	7.61	0.000104	0.00000533	4.07E-08
cAMP signaling pathway	21	0.000000395	0.00000799	3.89	0.000112	0.00000533	9.36E-08
Pancreatic secretion	14	0.000000793	0.000015	5.74	0.000224	0.00000999	0.000000122
Hormone signaling	20	0.00000106	0.0000181	3.82	0.000299	0.0000121	0.000000254
Thyroid hormone signaling pathway	15	0.00000109	0.0000181	5.15	0.000308	0.0000121	0.000000189
Pentose and glucuronate interconversions	9	0.00000154	0.0000236	10.46	0.000436	0.0000158	0.000000116
PPAR signaling pathway	12	0.00000159	0.0000236	6.61	0.000449	0.0000158	0.000000208
Amphetamine addiction	11	0.00000472	0.0000668	6.67	0.00134	0.0000446	0.000000611

Table S4. Top 10 GO enrichment data for inflammation-related targets.

Category	Term	Count	P-Value	Benjamini	Fold Enrichment	Bonferroni	FDR	Fisher Exact
GOTERM_BP_ DIRECT	G protein-coupled receptor signaling pathway, coupled to cyclic nucleotide second messenger	15	2.33E-16	5.68E-13	26.63	5.41E-13	5.24E-13	6.56E-18
GOTERM_BP_ DIRECT	response to lipopolysaccharide	18	2.18E-13	2.65E-10	11.74	5.31E-10	2.45E-10	1.66E-14
GOTERM_BP_ DIRECT	response to hypoxia	19	5.29E-13	4.29E-10	10.12	1.29E-09	3.96E-10	4.73E-14
GOTERM_BP_ DIRECT	response to xenobiotic stimulus	21	2.68E-12	1.63E-09	7.86	6.52E-09	1.5E-09	3.15E-13
GOTERM_BP_ DIRECT	G protein-coupled receptor signaling pathway	39	7.85E-12	3.82E-09	3.61	1.91E-08	3.53E-09	2.1E-12

GOTERM_BP_DIRECT	response to nicotine	11	9.81E-12	3.98E-09	25.72	2.39E-08	3.67E-09	2.9E-13
GOTERM_BP_DIRECT	inflammatory response	26	1.85E-11	6.43E-09	5.35	0.000000045	5.93E-09	3.28E-12
GOTERM_BP_DIRECT	positive regulation of MAPK cascade	19	2.71E-11	8.26E-09	8.02	6.61E-08	7.62E-09	3.12E-12
GOTERM_BP_DIRECT	chemical synaptic transmission	19	3.9E-11	1.06E-08	7.85	9.51E-08	9.75E-09	4.61E-12
GOTERM_BP_DIRECT	positive regulation of ERK1 and ERK2 cascade	18	7.14E-11	1.74E-08	8.22	0.000000174	0.000000016	8E-12
GOTERM_CC_DIRECT	plasma membrane	109	3.41E-13	1.03E-10	1.84	1.03E-10	9.06E-11	1.83E-13
GOTERM_CC_DIRECT	dendrite	24	1.46E-09	0.000000152	4.74	0.000000442	0.000000134	2.95E-10
GOTERM_CC_DIRECT	membrane	101	1.51E-09	0.000000152	1.69	0.000000455	0.000000134	8.81E-10
GOTERM_CC_DIRECT	RNA polymerase II transcription regulator complex	14	3.62E-09	0.000000273	9.32	0.00000109	0.00000024	3.55E-10
GOTERM_CC_DIRECT	extracellular matrix	22	1.16E-08	0.0000007	4.65	0.0000035	0.000000617	2.38E-09
GOTERM_CC_DIRECT	signaling receptor complex	14	0.000000255	0.0000113	6.51	0.000077	0.00000996	3.67E-08
GOTERM_CC_DIRECT	apical plasma membrane	19	0.000000265	0.0000113	4.48	0.0000801	0.00000996	5.66E-08
GOTERM_CC_DIRECT	synapse	26	0.000000299	0.0000113	3.31	0.0000904	0.00000996	8.73E-08
GOTERM_CC_DIRECT	postsynaptic membrane	13	0.00000689	0.000231	5.29	0.00208	0.000204	0.00000123
GOTERM_CC_DIRECT	cytoplasm	99	0.00000967	0.000292	1.44	0.00292	0.000257	0.00000661
GOTERM_MF_DIRECT	nuclear receptor activity	12	7.61E-12	4.83E-09	21.25	4.83E-09	4.06E-09	2.87E-13
GOTERM_MF_DIRECT	endopeptidase activity	14	4.15E-11	1.32E-08	13.26	2.63E-08	1.11E-08	2.74E-12
GOTERM_MF_DIRECT	G protein-coupled receptor activity	33	3.87E-10	0.000000082	3.65	0.000000246	0.000000069	1.02E-10
GOTERM_MF_DIRECT	steroid binding	9	7.67E-10	0.000000122	27.77	0.000000486	0.000000102	2.06E-11
GOTERM_MF_DIRECT	serine-type endopeptidase activity	16	1.54E-09	0.000000195	8.01	0.000000973	0.000000164	1.78E-10
GOTERM_MF_DIRECT	enzyme binding	22	2.65E-09	0.000000281	5.06	0.00000168	0.000000236	4.98E-10
GOTERM_MF_DIRECT	oxidoreductase activity, acting on single donors with incorporation of molecular oxygen, incorporation of two atoms of oxygen	7	3.3E-09	0.000000299	47.82	0.00000209	0.000000252	3.97E-11
GOTERM_MF_DIRECT	oxidoreductase activity	18	1.49E-08	0.00000118	5.80	0.00000946	0.000000996	2.43E-09
GOTERM_MF_DIRECT	flavin adenine dinucleotide binding	10	2.65E-08	0.00000187	14.49	0.0000168	0.00000157	1.58E-09
GOTERM_MF_DIRECT	neurotransmitter receptor activity	8	4.04E-08	0.00000256	23.19	0.0000256	0.00000216	1.38E-09

Table S5. Top 20 KEGG pathway enrichment data for oxidative stress-related targets associated with Anti-inflammatory activity.

Term	Count	P-Value	Benjamini	Fold Enrichment	Bonferroni	FDR	Fisher Exact
Neuroactive ligand signaling	31	1.26E-18	3.5E-16	7.85	3.5E-16	2.1E-16	1.39E-19
Chemical carcinogenesis - receptor activation	27	9.44E-14	1.32E-11	6.27	2.64E-11	7.89E-12	1.35E-14

Neuroactive ligand-receptor interaction	34	1.8E-13	1.67E-11	4.63	5.02E-11	1E-11	3.59E-14
Serotonergic synapse	19	8.06E-12	5.62E-10	8.33	2.25E-09	3.36E-10	8.27E-13
AGE-RAGE signaling pathway in diabetic complications	17	1.02E-10	5.7E-09	8.48	2.85E-08	3.41E-09	1.03E-11
Pathways in cancer	36	2.04E-10	9.5E-09	3.40	0.000000057	5.69E-09	5.67E-11
Calcium signaling pathway	24	8.73E-10	3.48E-08	4.76	0.000000244	2.08E-08	1.68E-10
Bile secretion	14	1.97E-08	0.000000686	7.84	0.00000549	0.000000411	2.17E-09
Insulin resistance	14	0.000000202	0.00000626	6.47	0.0000563	0.00000375	2.77E-08
MAPK signaling pathway	22	0.000000431	0.000012	3.70	0.00012	0.00000719	0.000000109
HIF-1 signaling pathway	13	0.00000159	0.0000404	5.96	0.000444	0.0000242	0.00000024
PPAR signaling pathway	11	0.00000217	0.0000504	7.30	0.000605	0.0000302	0.00000026
TNF signaling pathway	13	0.00000368	0.0000789	5.51	0.00103	0.0000472	0.000000604
Pentose and glucuronate interconversions	8	0.00000535	0.0000996	11.20	0.00149	0.0000596	0.000000389
Lipid and atherosclerosis	17	0.00000535	0.0000996	3.97	0.00149	0.0000596	0.00000125
Relaxin signaling pathway	13	0.00000925	0.000161	5.04	0.00258	0.0000965	0.00000167
Cholinergic synapse	12	0.0000157	0.000258	5.26	0.00437	0.000154	0.00000271
Hepatitis B	14	0.0000191	0.000296	4.33	0.00532	0.000177	0.00000409
Inflammatory mediator regulation of TRP channels	11	0.0000239	0.000351	5.60	0.00665	0.00021	0.00000385
EGFR tyrosine kinase inhibitor resistance	10	0.0000259	0.000359	6.30	0.00721	0.000215	0.00000367

Table S6. Grid-center coordinates, grid dimensions, and key AutoDock Vina parameters for STAT3, JUN, ESR1, CXCL8, and PTGS2.

Target	PDB ID	Chain	Resolution (Å)	Grid center (x, y, z)	Grid size (Å)	Exhaustiveness	Energy_range	RMSD (Å)
STAT3	6njs	A	2.7	x = 10.795<br>y = 55.352<br>z = 1.913	26.25	8	3	0.271
JUN	6y3v	A	1.5	x = 20.066<br>y = -14.824<br>z = -7.902	22.5	8	3	0.798
ESR1	3uud	A	1.6	x = 20.392<br>y = 2.158<br>z = 4.601	22.5	8	3	0
CXCL8	6lfo	R	3.4	x = 131.366<br>y = 124.833<br>z = 149.125	26.25	8	3	0.001
PTGS2	5f19	A	2.04	x = 27.446<br>y = 32.33<br>z = 60.255	26.25	8	3	0
